# Iodinated cyanine dye-based nanosystem for synergistic phototherapy and hypoxia-activated bioreductive therapy

**DOI:** 10.1080/10717544.2021.2023701

**Published:** 2022-01-08

**Authors:** Yunxia Dong, Ling Zhou, Zijun Shen, Qingming Ma, Yifan Zhao, Yong Sun, Jie Cao

**Affiliations:** a Department of Pharmaceutics, School of Pharmacy, Qingdao University, Qingdao, China; b The Key Laboratory of Traditional Chinese Medicine Prescription Effect and Clinical Evaluation of State Administration of Traditional Chinese Medicine, School of Pharmacy, Binzhou Medical University, Yantai, China

**Keywords:** Photodynamic therapy, hypoxia-activated chemotherapy, immune response, iodinated-cyanine dyes

## Abstract

Photodynamic therapy (PDT) has been applied in cancer treatment by utilizing reactive oxygen species (ROS) to kill cancer cells. However, the effectiveness of PDT is greatly reduced due to local hypoxia. Hypoxic activated chemotherapy combined with PDT is expected to be a novel strategy to enhance anti-cancer therapy. Herein, a novel liposome (LCT) incorporated with photosensitizer (PS) and bioreductive prodrugs was developed for PDT-activated chemotherapy. In the design, CyI, an iodinated cyanine dye, which could simultaneously generate enhanced ROS and heat than other commonly used cyanine dyes, was loaded into the lipid bilayer; while tirapazamine (TPZ), a hypoxia-activated prodrug was encapsulated in the hydrophilic nucleus. Upon appropriate near-infrared (NIR) irradiation, CyI could simultaneously produce ROS and heat for synergistic PDT and photothermal therapy (PTT), as well as provide fluorescence signals for precise real-time imaging. Meanwhile, the continuous consumption of oxygen would result in a hypoxia microenvironment, further activating TPZ free radicals for chemotherapy, which could induce DNA double-strand breakage and chromosome aberration. Moreover, the prepared LCT could stimulate acute immune response through PDT activation, leading to synergistic PDT/PTT/chemo/immunotherapy to kill cancer cells and reduce tumor metastasis. Both *in vitro* and *in vivo* results demonstrated improved anticancer efficacy of LCT compared with traditional PDT or chemotherapy. It is expected that these iodinated cyanine dyes-based liposomes will provide a powerful and versatile theranostic strategy for tumor target phototherapy and PDT-induced chemotherapy.

## Introduction

Photodynamic therapy (PDT), has been applied in the early diagnosis and treatment of various superficial tumors due to the unique advantages including long-range, controllable, selective, and systemic low toxicity (Cheng et al., 2014; Li et al., 2017a,b; Lee et al., 2021). In addition, PDT is reported to trigger the local acute inflammatory response of irradiated tissues, resulting in the rapid local infiltration of neutrophils and macrophages as well as the release of systemic inflammatory mediators (Li et al., 2018a,b; Zhao et al., 2021). It can also cause specific T lymphocyte activation, affecting the further development of inflammation to produce a large number of cytokines, adhesion molecules, co-stimulatory molecules, and immune-related important media, which ultimately control the growth of residual tumor cells (Wachowska et al., 2015; Donohoe et al., 2019; Udartseva et al., 2019; He et al., 2020). However, the natural hypoxic-tumor microenvironment is a major obstacle to the photodynamic reaction, limiting its applications in anoxic tumors (Liu et al., 2019, 2020; Shen et al., 2021; Yu et al., 2021). In addition, PDT-mediated oxygen consumption and microvessel injury further aggravate the anoxia of the tumor, which may lead to a variety of adverse effects, such as tumor invasion and metastasis (Zheng et al., 2008; Poderys et al., 2020).

In the past five decades, anticancer drugs have been used extensively in cancer therapy. However, tumor hypoxia cells show distinct resistance to conventional chemotherapeutic agents, which results in incomplete therapeutic efficacy and tumor recurrence (Zhang et al., 2018; Nagata et al., 2020). Hypoxic reactive drugs, such as tirapamide (TPZ), TH-302, PR-104A, and benzoate (AQ4N), have been demonstrated to provide high select cytotoxicity toward hypoxic mammalian cells (Zhang et al., 2018; Deng et al., 2019; He et al., 2019; Li et al., 2019; Ma et al., 2019; Chen et al., 2021). Among them, tirapazamine (TPZ) is a novel class of bioreductive drugs that can be metabolized to produce free radicals in the presence of anoxic cells, resulting in DNA damage and cell death. Currently, it is often used as a radiosensitizer and combined with cisplatin to treat tumors in the head, neck, lungs, and throat (Kaneda et al., 2019; Wang et al., 2019; Zhao et al., 2020). However, when used alone, the anti-tumor effect of TPZ is largely limited due to the insufficient toxic substances production in oxygen-relative enriched tumor cells in the vicinity of the tumor vessel (Baker et al., 2013). Therefore, creating a hypoxia environment in tumor sites is essential for enhancing the anticancer efficacy of TPZ.

Fortunately, despite the greatly reduced therapeutic efficacy of PDT in hypoxic microenvironments, the induced hypoxia can be exploited for the activation of bioreductive precursors, to overcome the therapeutic resistance of the hypoxic tumors (Broekgaarden et al., 2017; Song et al., 2020; Chou et al., 2021). Unlike other strategies to relieve tumor anoxia, these bioreductive precursors can not only take advantage of the hypoxic microenvironment of the tumor itself but also make use of the unfavored hypoxic conditions to increase the effect of hypoxia-activated drugs (Liu et al., 2015; Zhang et al., 2018). Recent researchers have proved the feasibility and effectiveness of this method by combining the delivery of photosensitizers and prodrugs in nanosystems, such as silica-shell nanoparticles or liposomes (Liu et al., 2015; Wang et al., 2017). However, most of the current strategies lack deep tissue penetration or low singlet oxygen (^1^O_2_) quantum yield of PS, resulting in insufficient oxygen-deficit environments.

**Scheme 1.**
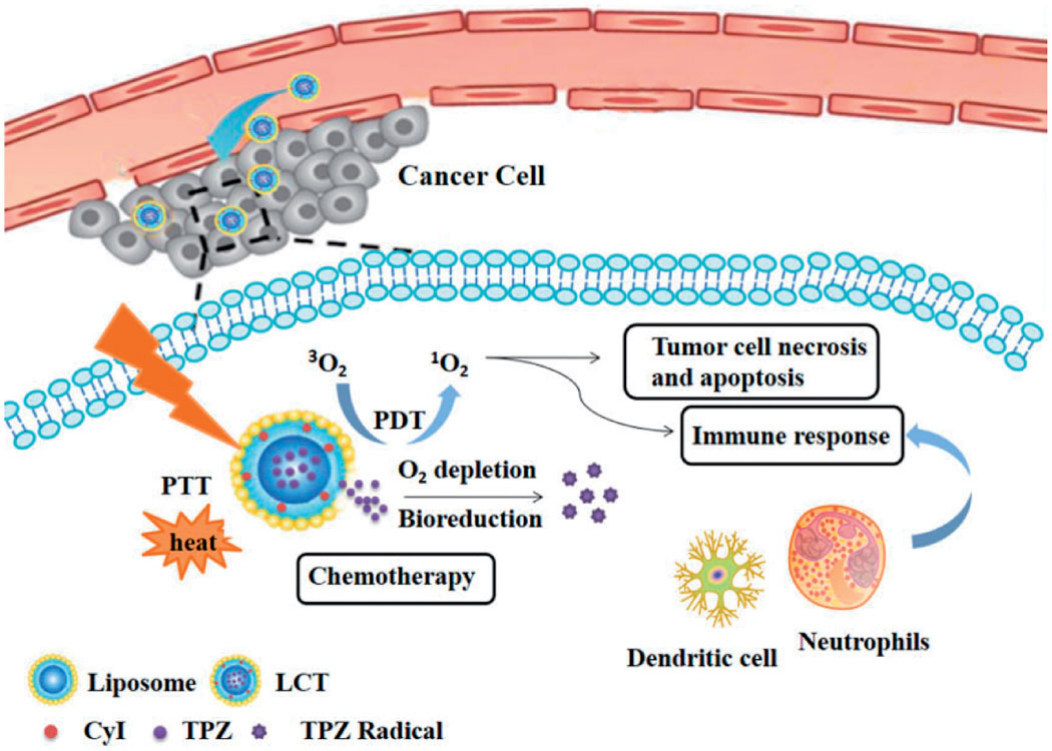
Scheme of LCT for highly efficient synergistic PDT/PTT/immunotherapy combined with hypoxia activated chemotherapy.

The quantity of ^1^O_2_ produced by photosensitizers is monitored by the efficiency of intersystem crossing (ISC), which indicates the spin-forbidden electronic transition from a singlet to a triplet state. The introduction of heavy atoms, such as Br, I into a molecule can influence the rates of ISC (Gorman et al., 2004; Lim et al., 2011; Zhou et al., 2012; De Simone et al., 2018; Shen et al., 2020). Indocyanine green (ICG) dyes, the only near-infrared (NIR) dye approved by the FDA (Vahrmeijer et al., 2013), exhibit absorbance in the NIR region, which is a transparency window for biological tissues and could efficiently transfer the absorbed NIR optical energy into fluorescence, ROS, and heat. However, the efficiency of directly using ICG as photosensitizer was very low, since only a very small percentage of the excited ICG can pass through the triplet decay that can produce ROS. In our previous work, an iodinated-ICG-derivative CyI with singlet oxygen quantum yield (Φ_Δ_) of 75% was synthesized and used for highly efficient NIR-guided synergistic phototherapy for the first time (Cao et al., 2019). Hence, in this study, we developed liposomes co-loaded with CyI and TPZ, which was termed as LCT for PDT-induced hypoxia chemotherapy (Scheme 1). After intravenous injection, such liposomes were expected to accumulate in tumor sites. Upon NIR irradiation, CyI loaded in LCT could simultaneously generate ROS and heat for combinatorial PDT/PTT, as well as provide fluorescence signals for precise real-time imaging. Meanwhile, due to the continuous consumption of oxygen by the PDT, the oxygen-deficient microenvironment can be locally created, activating hypoxia-responsive TPZ to selectively kill hypoxic malignant tumor cells, thus achieving synergistic antitumor effects. In addition, PDT-induced local acute inflammation and immune response can further lead to synergistic PDT/PTT/chemo/immunotherapy to kill cancer cells and reduce tumor metastasis.

## Materials and methods

### Materials

CyI (MW 776.5) was synthesized in our laboratory and TPZ was obtained from Beino Biotechnology Co. (Shanghai, China). Soybean phosphatidylcholine (PC) and cholesterol were acquired from TCI (Shanghai, China). N-(Carbonyl-methoxypolyethyleneglycol 2000)-1,2-distearoyl-sn-glycero-3-phosphoethanolamine (DSPE-mPEG_2000_) was purchased from Xiebao Bio-tech Co. (Shanghai, China). Singlet Oxygen Sensor Green (SOSG) was obtained from Invitrogen-Life Technologies (Carlsbad, CA, USA). Nuclear staining dye (Hoechst 33342), Calcein AM/PI assay kit, Methyl thiazolyltetrazolium (MTT), Annexin V-FITC/PI apoptosis staining kits were acquired from Solarbio (Beijing, China). Hypoxyprobe™-1 Plus Kits were obtained from Hypoxyprobe Inc. (USA). Hypoxia/Oxidative Stress Detection Kit was obtained from Enzo Life Sciences (USA). ELISA test kits of IFN-γ, IL-10, IL-12, TNF-α were purchased from Biolegend (San Diego, CA, USA). Immune factors were detected by an enzyme label analyzer (RaytoRT-6100, Rayto Co. Ltd., China).

4T1 cell was obtained from Shanghai Gefan Biotechnology Co. Ltd. cells were cultured in Dulbecco's Modified Eagle Medium (DMEM, Hyclone, USA) with 10% fetal bovine serum (Hyclone, USA) and 1%penicillin-streptomycin (Hyclone, USA). Cells were maintained at standard normoxic culture conditions (21% O_2_, 5%, 37 °C) or oxygen-deficient environments (<1% O_2_, 5% CO_2_, 37 °C)using a gas mixture of 95% nitrogen, 5% CO_2_ (Linde Gas, Schiedam, the Netherlands). BALB/c mice (seven weeks old, 15–18 g) were obtained from Daren Laboratory Animal Co. Ltd. (Qingdao, China). The mice used in the experiment were treated according to the scheme approved by the Chinese ethics committee.

### Synthesis of LCT

The liposomes encapsulating CyI and TPZ were synthesized by a thin-film hydration method. In short, lecithin, cholesterol, DSPE-mPEG_2000_, and CyI were dissolved in methanol at specific concentrations and then remove the organic solvent. Next, 10 mL PBS containing TPZ was added to dissolve the film under vortexing for 1 h at 45 °C. The resulting mixture was subjected to ultrasound at 250 W for 5 min, and finally, the un-encapsulated CyI and TPZ were removed by 0.22 μm nitrocellulose membrane.

### Characterization

The particle diameter of LCT was determined by a dynamic light scattering Zetasizer (Nano ZS, Malvern, UK). The shape and size of the LCT were visualized by TEM (JEOL, JEM-1200EX). Simultaneously, the particle size stability of LCT was evaluated by placing it in water, PBS, fetal bovine serum (FBS), and medium (10% FBS) for five weeks at room temperature. The absorption and fluorescence spectra were detected by UV–vis spectrophotometry (Beckman Coulter DU 640) and Fluorolog-3 fluorescence spectrum, respectively. Drug loading content and encapsulation of LCT were analyzed by ultracentrifugation and the transmission method. The LCT was firstly centrifuged in the ultrafiltration tube at 10,000 rpm for 30 min. The free TPZ enters the centrifuge tube through the ultrafiltration tube, simultaneously free CyI is filtered by the transmission method, while the total amount of TPZ and CyI is acquired by the emulsification method. The drug loading content of LCT was calculated according to Equation (1):

(1) Drug loading (%)=the loaded drug mass/the total mass of LCT×100%

Concurrently, the encapsulated efficiency of LCT was calculated according to Equation (2):

(2) Encapsulated efficiency (%)=the loaded drug mass/total TPZ or CyI×100%

### NIR light-triggered drug release

To assess the release behavior of CyI and TPZ, LCT (CyI, 125 μg/mL; TPZ, 100 μg/mL; 2 mL) were placed in a dialysis box filled with PBS (37 °C) at pH 7.4 with and without NIR light at 0.96 W/cm^2^ for 100 min. The TPZ and CyI content outside the dialysis bag was detected using UV–VIS spectrophotometry. Meanwhile, the drug release in liposomes was further evaluated by TEM (JEOL, JEM-1200EX) for 30 s, 1, 3, 5, and 10 min.

### Measurement of dissolved oxygen

Briefly, add LCT (CyI, 125 μg/mL) to the beaker and seal it with liquid paraffin to block the oxygen flow. Then, the oxygen concentration of LCT was recorded using a dissolved oxygen meter every 30 s with or without NIR irradiation.

### Measurement of *in vitro* photodynamic and photothermal effects

^1^O_2_ was detected by specific probe SOSG, a ^1^O_2_ probe whose fluorescence will be increased after reacted with^1^O_2_. Briefly, 150 µL PBS, LC, LCT, or CyI (125 μg/mL) was mixed with 50 µL SOSG (25 μM), then the samples were subjected to 808 nm laser under different power densities for different times (808 nm, 0.3, 0.96, or 1.6 W/cm^2^, 1–7 min), and the SOSG fluorescence was detected immediately using Flex Station 3 (Ex: 504 nm/Em: 525 nm).

The photothermal effect of LCT was also evaluated by recording the temperature curve at different power irradiation (0.3, 0.96, or 1.6 W/cm^2^) as the irradiation time increases. The curve of the temperature over time for blank Lipid, LT, LC, and LCT (125 μg/mL) at 0.96 W/cm^2^ were also evaluated. Moreover, photothermal effects were also assessed by irradiation of different concentrations of LCT (0.96 W/cm^2^, 6 min). Temperatures were quantified using a thermocouple thermometer (TES-1310) at every 30 s. These results were recorded by a thermal imager (Xtherm T3Pro, IRay, China) at the highest temperature.

### Cell uptake

The uptake of LCT into cells was studied by confocal microscopy and flow cytometry. First, 4T1 cells were cultured in a dish for 24 h. The medium was then sucked out and cultured with LCT (125 µg/mL, 2 mL). After incubation for 1, 2, 4, and 8 h, respectively, wash the dish twice with PBS and stain with Hoechst33342 for 15 min. Then it was washed three times with PBS and photographed with the confocal method. Meanwhile, after treatment with the LCT for 1, 2, 4, and 8 h, respectively, the cells were digested, collected, and analyzed by flow cytometry.

### Intracellular ROS/hypoxia assay

The generation of ROS and anoxia in cells was assessed by Hypoxia/oxidative stress kit, 4T1 cells were cultured in a dish for 24 h and set up five groups. The medium was then sucked out and cultured with (1) PBS; (2) fresh medium and irradiated 5 min with 808 nm (0.96 W/cm^2^) laser only; (3) TPZ; (4) LC with NIR light (0.96 W/cm^2^) irradiation for 5 min; (5) LCT with IR light (0.96 W/cm^2^) irradiation for 5 min. Besides, the medium was then sucked out and cultured with anoxia inducer (DFO) for 3 h or with ROS inducer (Pyo) for 0.5 h, respectively. After 4 h of incubation, the detection probe was cultured for 5 min and observed by confocal.

### Intracellular photothermal effect detection

The intracellular photothermal effect was evaluated by temperature variation. Firstly, treating 4T1 cells with blank Lipid, LT, or LCT for 4 h, then cells were collected by centrifugation and exposed to a NIR laser for 6 min (808 nm, 0.96 W/cm^2^). The rise in cell temperature was recorded every 30 s by a thermocouple thermometer and thermal images were recorded using an infrared thermal imaging camera (Xtherm T3Pro, IRay, China). The photothermal effect of LCT was also evaluated by recording the temperature curve with NIR light at different power irradiation (0.3, 0.96, or 1.6 W/cm^2^).

### *In vitro* cytotoxicity studies

#### MTT assay

The cytotoxicity of 4T1 cells was studied by the MTT experiment. Firstly, the 4T1cells were cultured in the 96-well plate at the density of 1 × 10^5^ per well for 24 h. Then remove the medium and replace it with different concentrations of LC. After 4 h incubation, irradiating cells with NIR light for 5 min or without laser. Then continue to culture for 24 h and add 10 µL MTT (5 mg/ml) accordingly. After 4 h, 150 µL dimethyl sulfoxide was added, and the absorbance was detected by a microplate reader at 570 nm.

In addition, 4T1 breast cancer cells were cultured in the 96-well plate under normal and hypoxic conditions, then remove the medium and replace with different concentrations of TPZ, and the same detection is then performed. Afterward, 4T1 breast cancer cells were cultured in the 96-well plates under hypoxic conditions. After 24 h, treating 4T1 cells with various samples (PBS, NIR, TPZ, LT, LC + 0.96 W/cm^2^, LCT + 0.3 W/cm^2^, LCT + 0.96 W/cm^2^) was added to the plate. After 4 h, irradiating cells with NIR light for 5 min or without laser, then the above tests are carried out.

#### Live/dead cell staining assay and cell apoptosis

Live-dead cell staining tests are used to detect the ability to kill 4T1 cells. Firstly, Cells were cultured in a petri dish for 24 h. After that, the cells were cultured with PBS, NIR, TPZ, LC, LCT, and irradiated with NIR light (808 nm, 0.3 W/cm^2^, or 0.96 W/cm^2^) for 5 min after culture for 4 h. After 24 h, the cultured cells were stained with AM (4 × 10^−6 ^M) and PI dye (4 × 10^−6 ^M) for 15 min, then washed with PBS three times and examined by a confocal microscope.

At the same time, apoptosis was detected by the flow of the annexin V-FITC/PI kit. The cells were cultured in a petri dish for 24 h and the sample was added as above. Then, irradiated with NIR light (0.3 or 0.96 W/cm^2^) for 5 min. The apoptosis was detected by flow cytometry after 24 h of culture.

#### Hemolytic toxicity

The hemo-compatibility of LCT was evaluated in terms of the hemolysis ratio. LCT (62.5–500 µg/mL) was added to the red blood corpuscle suspension and the cell lysate Triton-X-100 was selected as the control. The mixture was cultured at 37° for 4 h and centrifuged at 3000 rpm for 20 min. After centrifugation, take the supernatant and measure its absorbance at 570 nm. The percentage of hemolysis was evaluated according to the absorbance factor of the 100% hemolytic sample Triton-X-100 in ultrapure water.

#### *In vivo* biodistribution and pharmacokinetics

First, 200 μl 4T1 cell suspension (1 × 10^6^ cells) was injected subcutaneously into the right leg of BALB/c mice. When the volume of the tumor increased to 250–300 mm^3^, the caudal vein was injected with LCT containing 1.2 mg/kg CyI and 0.96 mg/kg TPZ. The distribution of tumor-bearing mice was then taken at different time points after intravenous injection (1, 2, 4, 8, 12, and 24 h) by using an IVIS spectrum imaging system. After 24 h, the main organs and tumor sites of the mice were collected and photographed.

To detect the concentration of LCT in plasma and the distribution in tissue, the mice were intravenously injected with LCT (CyI, 1.2 mg/kg; TPZ, 0.96 mg/kg), and ∼30 µL of blood was collected at different time points (1, 2, 4, 6, 8, 12, and 24 h), then using 1 ml lysis buffer for ultrasonic lysis. Subsequently, the supernatant was obtained by centrifugation, 600 μL of supernatant was converged and dried overnight for UV–VIS spectrophotometer and HPLC. Meanwhile, the heart, liver, spleen, lung, and kidney were selected according to the predetermined time points. The tissues were dissolved into a cold saline solution and tissue homogenates were obtained by auto homogenizer (SCIENTZ-48, Ningbo Scientz Biotechnology, China). After centrifugation, the sediment was removed and analyzed by UV–VIS spectrophotometer and HPLC.

#### Evaluation of PDT/PTT effect *in vivo*

When the volume of the tumor increased to 250–300 mm^3^, saline, TPZ, LC, or LCT containing 1.2 mg/kg CyI and 0.96 mg/kg TPZ were intravenously injected. After 12 h, 50 μL SOSG (25 μM) was injected into the tumor and irradiated with NIR light for 5 min. Subsequently, the tumors were collected and frozen sections were made and shooting fluorescence by laser scanning confocal microscopy.

Meanwhile, to estimate the photothermal capacity *in vivo*, 4T1 tumor-bearing mice were intravenous injected separately with saline, TPZ, LC, and LCT at the CyI dose of 1.2 mg/kg. After 12 h, the tumor site of mice was irradiated with 808 nm NIR light at 0.96 W/cm^2^ for 5 min or without irradiation. At the same time, the temperature of the tumor surface was recorded every 30 s by thermocouple thermometer and thermal images were recorded by an infrared thermal imaging camera.

#### Hypoxia immunofluorescence staining

To detect hypoxia at the tumor site, 4T1 tumor-bearing mice were injected with Saline, TPZ, LC, or LCT containing 1.2 mg/kg CyI and 0.96 mg/kg TPZ. Then, the tumor site was irradiated with a NIR laser (0.96 W/cm^2^) for 5 min after 12 h. Subsequently, pimonidazole hydrochloride (60 mg kg^−1^) was injected intraperitoneally and the tumor was collected 90 min later, labeling the tumor hypoxia region with a fluorescein-conjugated IgGl antibody. After that, the sections were stained with anti-FITC secondary protocol and the fluorescence was observed by confocal microscope.

#### *In vivo* therapeutic efficacy

Firstly, 1 × 10^7^ 4T1 cells were subcutaneously injected into the mice's right leg. The length (*L*) and the width (*W*) of the tumor were measured by vernier caliper and the volume was calculated by the following formula: Tumor (mm^3^) = *L* × *W* × *W*/2. When the volume of the tumor increased to 250–300 mm^3^, treating with (1) saline, (2) NIR light, (3) free TPZ, (4) LC + 0.96 W/cm^2^, (5) LCT + 0.96 W/cm^2^, and (6) LCT + tissue + 0.96 W/cm^2^ (containing 1.2 mg/kg CyI and 0.96 mg/kg TPZ) *via* intravenous administration.

The time of the first dose was counted as 0 day, the group requiring laser irradiation was given 808 nm NIR light (0.96 W/cm^2^, 5 min) after 12 h of administration and then repeat the treatment every 7 days. Tumor volume and body weight were recorded every two days during the period. After 21 days, the mice were sacrificed and collected tumors for further study. Representative tumors from each group of animals were collected for H&E staining.

#### PDT induced immune response

To evaluate PDT-induced acute inflammatory response and immune response, 4T1 tumor-bearing mice of each group were injected with Saline, TPZ, LC, or LCT. Then the 808 nm NIR light (0.96 W/cm^2^, 5 min) was conducted after 12 h of injection. Five days later, the tumors of each group of mice were collected for immunological detection. Firstly, the tumor sections were put into the glass homogenizer and used a homogenization mechanism to form a single-cell suspension. Then the tumor supernatant was collected and the levels of IL-10, IL-12, IFN-γ, TNF-α, were detected by ELISA assay (eBioscience). Meanwhile, Anti-CD3-APC and anti-CD8-PE (eBioscience) staining were used to detect CTL content in tumor tissues by flow cytometry.

### Statistical analysis

Data were expressed as mean ± standard deviation. Statistical analysis was performed by student's *t*-test with statistical significance assigned for a *p*-value of <.05.

## Results and discussion

### Synthesis and characterization of LCT

**Figure 1.**
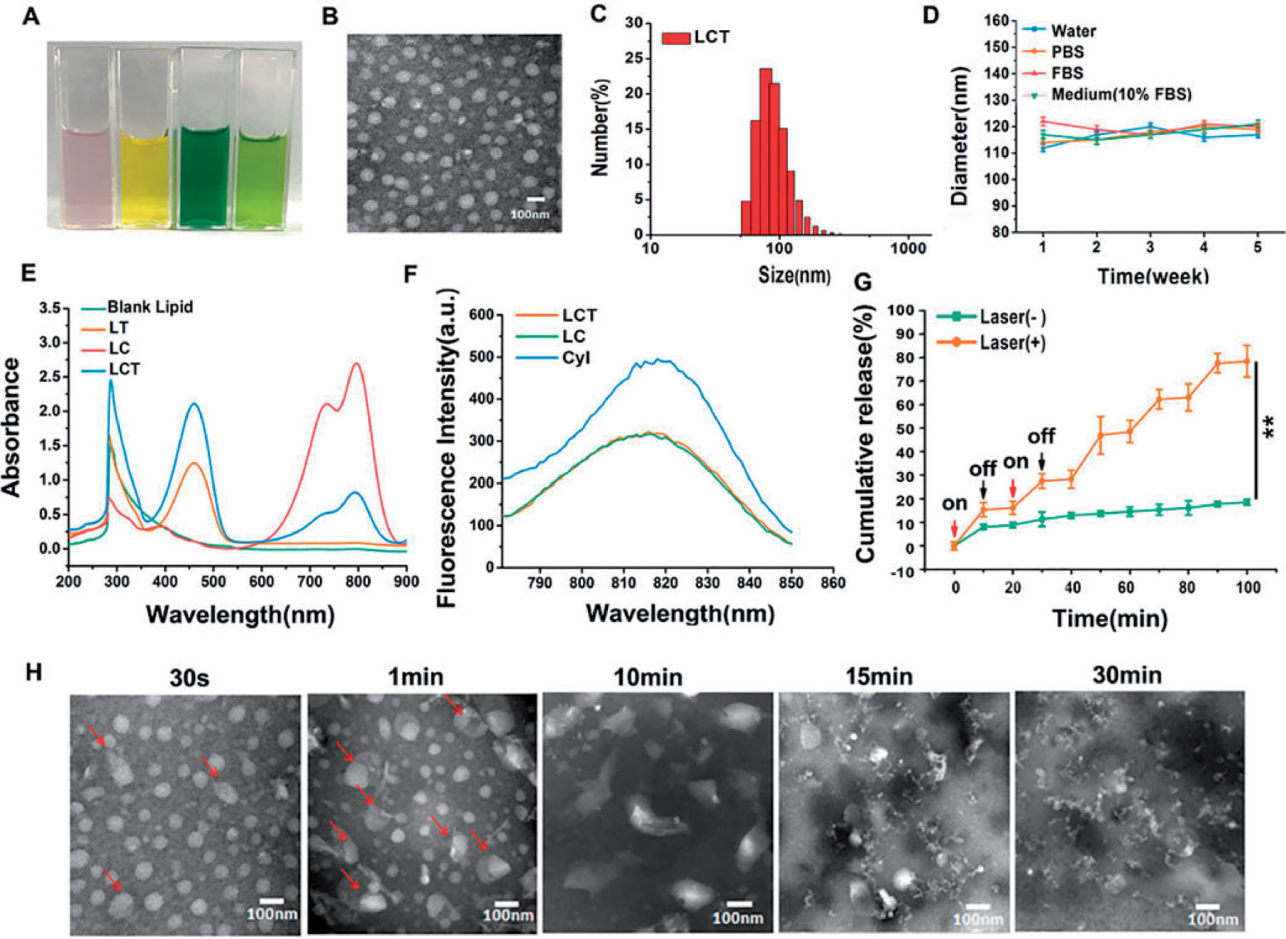
(A) Photographs of blank liposome, LC, LT and LCT aqueous solution; (B) TEM image of LCT (scale bar = 100 nm); (C) Hydrodynamic diameter of LCT; (D) Size stability of LCT stored in water, PBS, FBS, and DMEM medium (10% FBS) in 5 weeks; (E) Absorption spectra of blank liposome, LC, LT, and LCT; (F) Fluorescence spectra of CyI, LC and LCT; (G) Cumulative TPZ release curve from LCT with or without an ‘on-off’ NIR light (0.96 W/cm^2^) for 100 min at 37 °C (*n* = 3). **p < .01; (H) TEM images of LCT continuously irradiated with an 808 nm laser (0.96 W/cm^2^) for different time.

To construct liposomes for PDT-induced chemotherapy, CyI, an iodinated cyanine dye as the photosensitizer/photothermal/NIR imaging agent, was integrated with the hypoxia-activated prodrug TPZ, and the two compounds were co-encapsulated into liposomes through film hydration to method to form LCT. In addition, DSPE-PEG_2000_ was used to improve liposome stability. As shown in Figure 1(A), the color of liposomes changed after co-loading TPZ and CyI. When blank liposome, LT, LC, and LCT dissolved in PBS, it displayed light pink, yellow, dark green, and grass green, respectively. After the synthesis, we first confirmed the structure of LCT via transmission electron microscope (TEM) and dynamic light scattering (DLS). The TEM image demonstrated that the liposomes are monodisperse, spherical, and uniform in size with 90 nm, as shown in Figure 1(B). Data from Dynamic Light Scattering (DLS) demonstrated the size of LCT measured as 149 ± 1.86 nm (Figure 1(C)) and the polydispersity index (PDI) valued as 0.18 ± 0.0149, which would be easier to penetrate deeper tumor areas and more likely to be internalized by tumor cells through the enhanced permeability and retention effect (EPR). The particle size determined by DLS was slightly higher than that of TEM because of the evaporation of water resulting in shrinkage of liposomes during the sample preparation of TEM. And the zeta potentials of LCT was −4.11 ± 1.48 mV. Furthermore, to evaluate the stability of LCT, the size of LCT in different media (water, PBS, FBS, and medium with 10% FBS, respectively) was monitored within 5 weeks (Figure 1(D)), the results demonstrate that the LCT have long-term stability without precipitation or phase separation in water, PBS, FBS and medium (10% FBS), with good stability during storage. Additionally, UV–Vis-NIR absorption spectra showed the absorption peak of LCT located at 460 and 790 nm, respectively (Figure 1(E)), corresponding to the absorption peaks of TPZ at 463 nm and CyI at 790 nm, suggesting the successful encapsulation of CyI and TPZ into the liposome. Then, the typical fluorescence spectra of CyI, LC, and LCT were explored (Figure 1(F)). The results showed LCT had the maximum fluorescence intensity at 820 nm, indicating that the liposomes containing CyI could be excited in the NIR region for real-time imaging. According to Equations (1) and (2), the encapsulation efficiency of TPZ and CyI was 65.58 ± 2.66 and 52.09 ± 2.19%, respectively; while the loading efficiency was calculated to be 5.99 ± 0.24 and 4.76 ± 0.2%, respectively.

The real-time release profile of TPZ and CyI from LCT was monitored by an impulse ‘on-off’ irradiation within 100 min and plotted in Figure 1(G) and S10. Results showed that upon NIR irradiation, the release rate of TPZ and CyI from LCT could reach up to 79.97 and 61.36%, respectively, much higher than that without laser irradiation. This can be explained by the hyperthermia produced from CyI could melt fatty acids of liposomes (melting point of the eutectic is 39 °C), leading to the quick release of bioreducing drug-TPZ. The NIR light-induced degradation of LCT was then monitored by TEM. As shown in Figure 1(H), when the liposomes are continuously irradiated by NIR light (0.96 W/cm^2^) for 1 min, the morphology of nanoparticles began to change, and most of the liposomes were aggregated into larger nanospheres after 10 min irradiation. After 15 min irradiation, the obvious collapse of the nano-objects was observed and the particles lost their morphological integrity after continuous irradiation for 30 min. These results proved our hypothesis that LCT can be used as NIR light-triggered nanocarriers for drug release.

**Figure 2.**
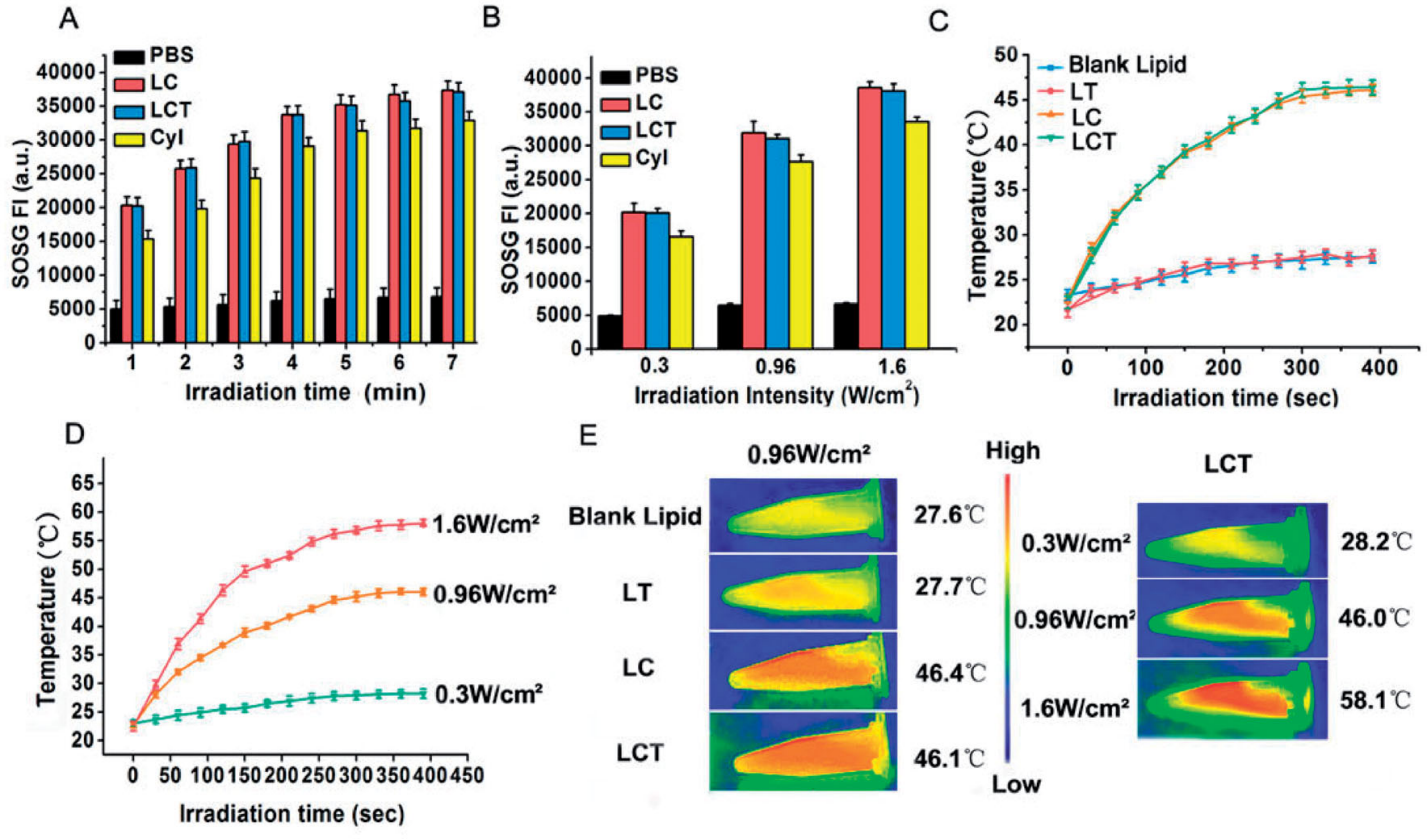
(A) Singlet oxygen production by PBS, LC, LCT, and CyI at different irradiation time (1–7 min) (*n* = 3); (B) Singlet oxygen production by PBS, LC, LCT, and CyI at different power density (0.3, 0.96, or 1.6 W/cm^2^, 5 min) (*n* = 3); (C) Temperature change curves of blank liposome, LC, LT, and LCT upon laser irradiation (0.96 W/cm^2^) (*n* = 3); (D) Temperature curves of LCT under different power density (0.3, 0.96, or 1.6 W/cm^2^) (*n* = 3); (E) Near infrared thermography of different samples under different power density (0.3, 0.96, or 1.6 W/cm^2^, 7 min).

CyI was confirmed to show excellent PDT/PTT properties in our previous study. Hence, to explore the phototherapy ability of LCT, the singlet oxygen and heat generation were evaluated. ^1^O_2_ generations of LCT were investigated by using SOSG as an indicator. SOSG is a singlet oxygen-specific fluorescent probe that can react with ^1^O_2_ and produce green fluorescence; therefore, we determined the ^1^O_2_ generation by detecting the fluorescence intensity at the corresponding wavelength. As shown by the results in Figure 2(A,B), obvious time and laser power-dependent enhancement of fluorescence intensity generated from LC and LCT were observed under laser irradiation (808 nm). The fluorescence intensity of LCT was comparable to that of LC and higher than that of CyI in aqueous solution, indicating that LCT has high singlet oxygen content and can be used for effective PDT treatment. In addition, the fluorescence intensity is stable after 5 min, illustrating that enough PDT effect could be obtained by using irradiation for 5 min. It should be mentioned that CyI is a hydrophobic dye and can easily form H-aggregate in water solution and thus result in reduced PDT efficacy. Our design has significantly improved the hydrophilicity of CyI, therefore enhancing singlet oxygen generation.

Upon NIR irradiation, CyI-mediated photodynamic therapy consumed oxygen to lead the oxygen-deficient microenvironment, which activated antitumor activity of the codelivered TPZ for synergistic cell-killing effect. Therefore, a dissolved oxygen meter was used to detect the changes in oxygen concentration in the PDT process. As shown in Figure S9, compared with the LCT group without NIR irradiation, the oxygen concentration of the LCT + NIR group continuously decreased during 5 min, indicating that the process of CyI-mediated photodynamic therapy-induced oxygen consumption.

The photothermal capacity of LCT was assessed by monitoring the temperature changes in aqueous dispersion. Temperature profiles of blank liposomes, LT, LC, and LCT upon 808 nm laser irradiation at 0.96 W/cm^2^ were firstly evaluated. As shown in Figure 2(C), the temperature of LC and LCT with 0.96 W/cm^2^ rapidly rose up during the irradiation, with the maximum temperature of 46.4 and 46.1 °C, respectively. In contrast, the temperature was slightly increased in the blank liposome and LT. Meanwhile, temperature profiles of LCT with different concentrations or power densities upon NIR irradiation for 5 min showed obvious concentration and laser power density dependence (Figure 2(D) and Figure S1). Interestingly, when the power density was lower than 0.3 W/cm^2^, no photothermal properties were observed, corresponding with our previous study of CyI. Then, a thermal image was used to record the maximum temperature of different samples at different power densities under NIR irradiation within 7 min (Figure 2(E)). The color change from yellow to red indicated that the temperature increased, which was consistent with results in Figure 2(C,D). Moreover, when the temperature rises to 43 °C, it can cause tumor thermal ablation. Therefore, all the results demonstrated that LCT has an excellent photothermal effect, and the PTT effect could be turned on or off by changing the irradiation laser power density.

### *In vitro* study

**Figure 3.**
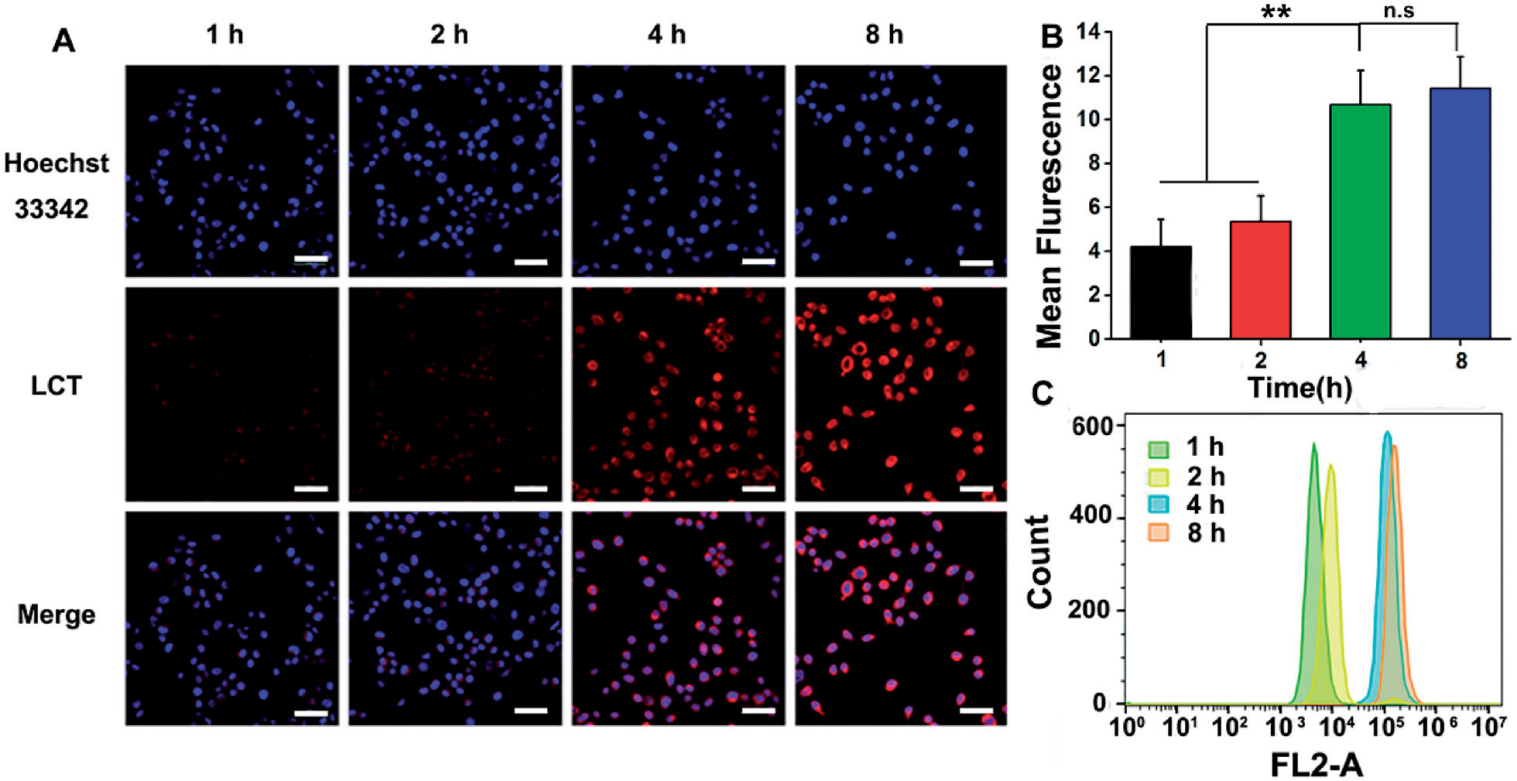
(A) Confocal fluorescence pictures of 4T1 cells cultured with LCT for 1, 2, 4, and 8 h, respectively. Scale bar is 50 μm; (B) Semi quantitative fluorescence analysis of 4T1 cells cultured with LCT for different time (*n* = 3). **p < .01, n.s.: not significant; (C) The flow cytometric analysis of cellular uptake in 4T1 cells cultured with LCT for different time.

To investigate the synergistic phototherapy and PDT-induced chemotherapy in cells, the intracellular uptake profiles were firstly studied. The behaviors of LCT against 4T1 cells were investigated by confocal laser scanning microscope (CLSM) and flow cytometry. As shown in Figure 3(A), cells were cultured with LCT for 1, 2, 4, and 8 h, respectively, and Hoechst 33342 was used to label cell nuclei. It can be seen that red fluorescence generated by LCT inside 4T1cells increased gradually along with the incubation time, suggesting that LCT internalized efficiently into 4T1 cells. The flow quantitative results in Figure 3(B) showed that the uptake of LCT in 4 h was significantly increased, compared with that of 1 or 2 h, and there was no significant difference from that of 8 h, illustrating that the LCT was almost internalized into 4T1 cells after 4 h incubation. Hence, we chose to apply NIR laser for cell irradiation after 4 h incubation in the following studies.

**Figure 4.**
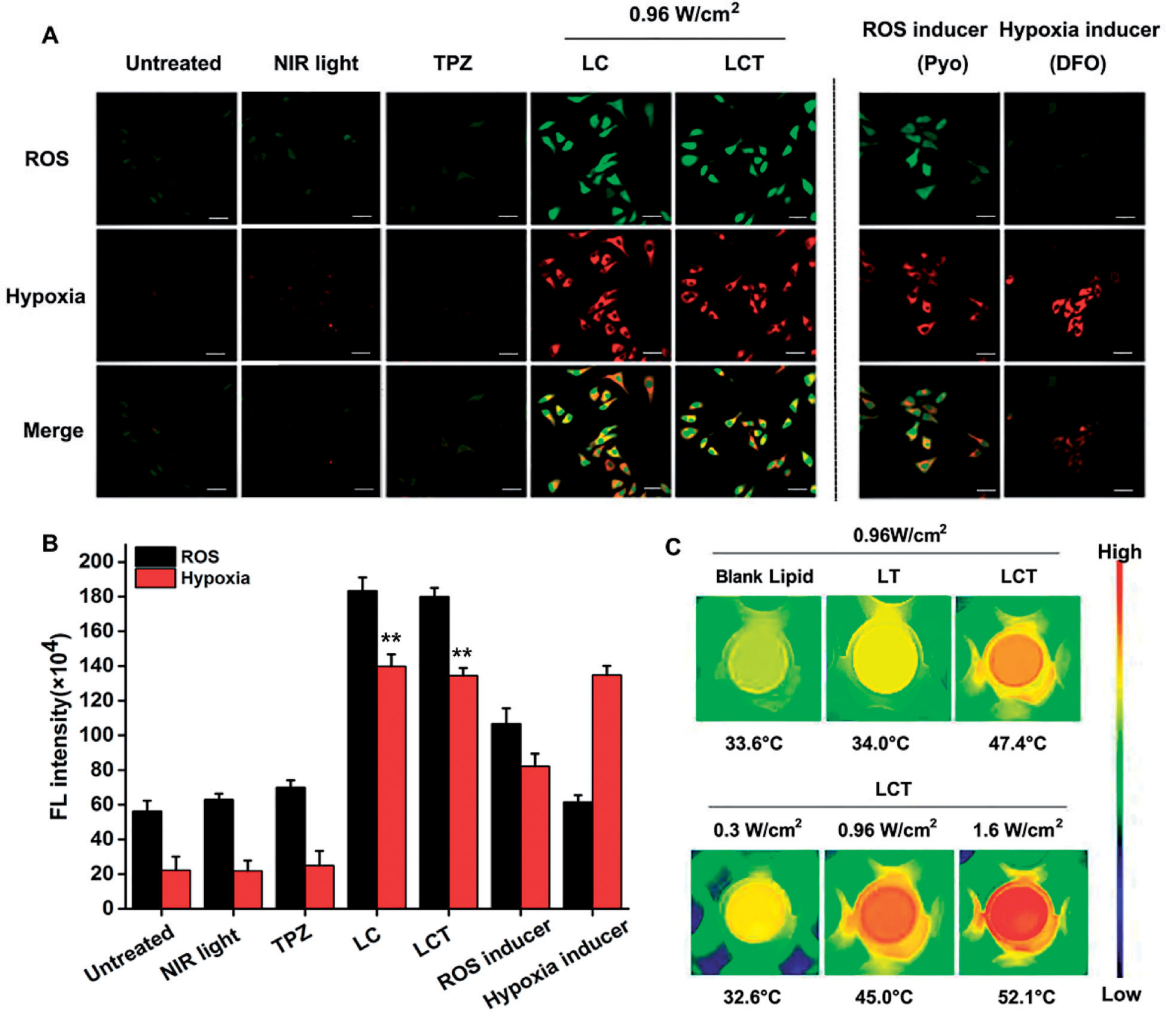
(A) Confocal fluorescence pictures of 4T1 cells cultured with TPZ, LC, or LCT upon NIR light (808 nm, 0.96 W/cm^2^) to assess ROS generation (green fluorescence) and anoxia conditions (red fluorescence). The untreated group and the NIR-only group were used as positive controls. Scale bar is 40 μm; (B) Semiquantitative analysis of ROS production and hypoxia (*n* = 3). **p < .01 *vs.* untreated group; (C) Thermal images of 4T1 cells under different laser irradiation after different treatments.

After confirming the optimized time for ingestion, the capability of LCT-mediated ROS generation and hypoxia condition was detected by a Hypoxia/Oxidative stress probe in living cells. Anoxic dyes are used to transform nitro into hydroxylamine (NHOH) and amino (NH_2_) by the activity of nitroreductase in anoxic cells and showed red fluorescence (596/670 nm), while the oxidative stress reagent is a kind of total reactive oxygen test agent which has cell permeability and can be directly related to reactive substances and showed green fluorescence (490/525 nm). As illustrated in Figure 4(A), negligible green and red fluorescence were observed in the absence of PS or laser irradiation, showing that the use of laser alone had little effect on ROS and hypoxia production. Similarly, the use of prodrug TPZ alone also did not produce ROS or hypoxia. In contrast, obvious green and red fluorescence was found in 4T1 cells incubated with LC or LCT in the presence of laser irradiation, indicating the substantial ROS generation of the LC and LCT, which further led to cell hypoxia. In addition, ROS inducers (Pyo) and hypoxia inducers (DFO) were used as positive controls to evaluate the amount of produced ROS and the degree of hypoxia. As shown in Figure 4(B), LC and LCT exhibited stronger green fluorescence compared to Pyo; while compared with DFO, the difference in red fluorescence was not distinct, which strongly indicated that LC and LCT could produce a large amount of ROS with excellent PDT effect, and at the same time the enhanced PDT could induce enough hypoxic environment for prodrug TPZ chemotherapy.

Then, the intracellular photothermal effect of LCT was studied by infrared thermal imaging camera and thermocouple thermometer under NIR irradiation. As shown in Figure 4(C), compared with the blank liposome and LT (0.96 W/cm^2^), the maximum temperature of LCT with 0.96 W/cm^2^ could reach 47.4 °C, which was much higher than the required value for irreversible apoptosis of tumor cells (43 °C). The temperature change curves were shown in Figure S2, respectively; the results are consistent with those of thermal images. After verifying the photothermal treatment ability of LCT, the temperature changes of LCT were then studied by changing power densities. As shown in Figure S3, a power-dependent temperature rise was observed, among which we choose the power of 0.96 W/cm^2^ as the treatment power for PTT. As expected, no PTT properties were monitored upon 0.3 W/cm^2^ irradiation. These results showed that the CyI-mediated heat generation of LCT not only had a good photothermal therapeutic ability but also could be adjusted from PDT to PDT/PTT combined therapy by changing the laser density from 0.3 to 0.96 W/cm^2^.

**Figure 5.**
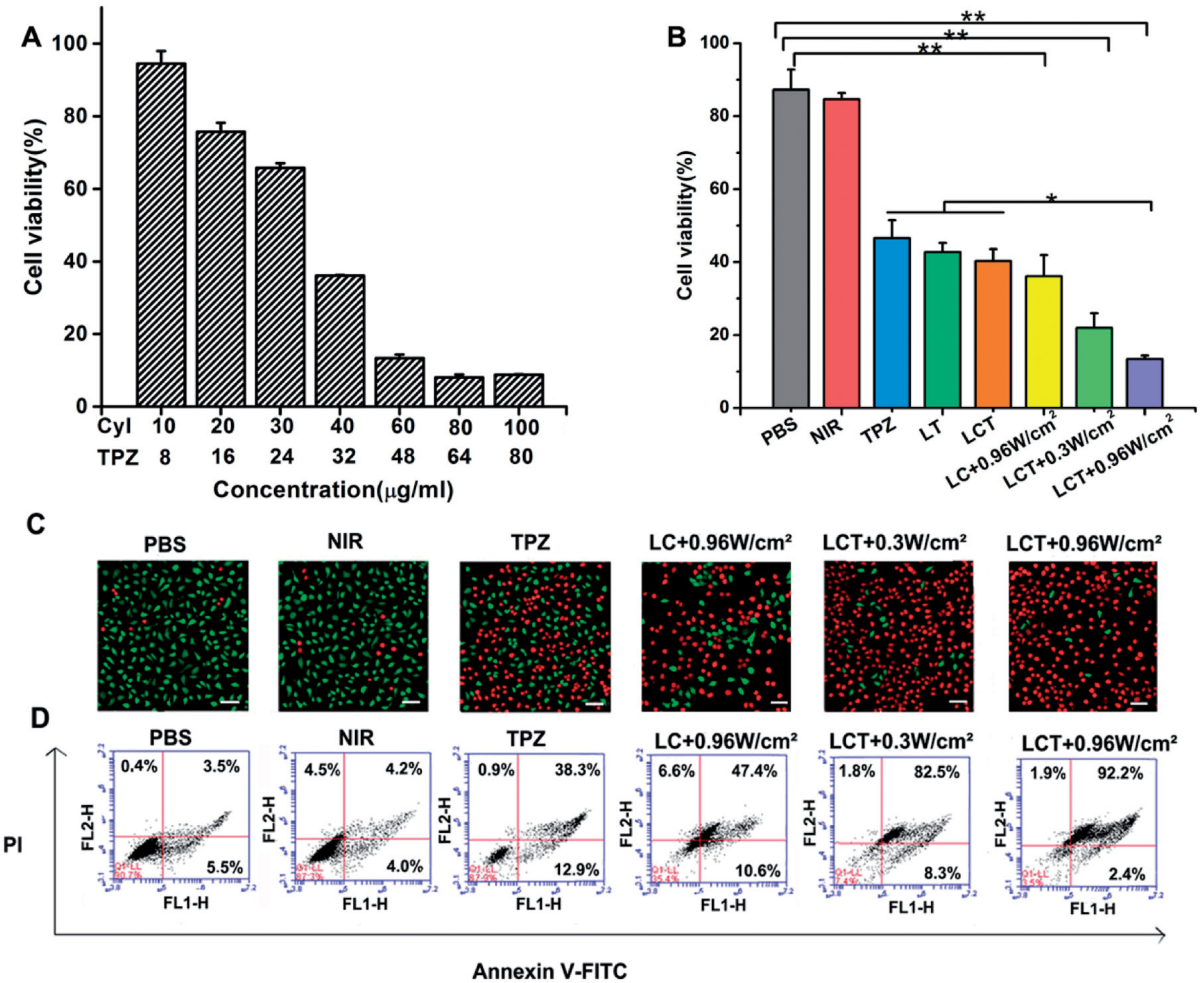
(A) The cell viability of 4T1 cells after incubation with different concentration of LCT under NIR light (808 nm, 0.96 W/cm^2^) (*n* = 3); (B) The cell viability of 4T1 cells at 24 h through various treatments (*n* = 3). *p < .05, **p < .01; (C) The living and dead cell staining of 4T1 cells through various treatments. Scale bar is 100 μm; (D) The cell apoptosis quantified by the flow cytometry at 24 h after various treatments.

The *in vitro* phototherapy and hypoxia-activated cytotoxicity of LCT was further investigated by the MTT experiment. Considering the tumor micro-environmental oxygen contents and the fact that PDT and hypoxia-reactive drugs were both oxygen-dependent, *in vitro* cytotoxicity of LT was studied in anoxic and normoxic 4T1 cells, respectively. As shown in Figure S4, LT exhibited a more efficient therapeutic ability toward hypoxic conditions (80.95 ± 2.4% cell death) than normoxic conditions (15.1 ± 3.22% cell death), indicating that TPZ has potent and selective cytotoxicity to hypoxia tumor cells. The phototherapy cytotoxicity of LC was also assessed in 4T1 cells. We discovered that cells cultured with LC followed by 808 nm light irradiation (0.96 W/cm^2^) could be efficiently killed (97.3 ± 2.4% cell death), while little inhibition rate in those incubated with LC without light irradiation (12.05 ± 5.3% cell death) (Figure S5). Those results confirmed effective PTT/PDT therapeutic efficacy upon NIR irradiation and hypoxia-activated cytotoxicity, which makes LCT have a great prospect in PDT-induced hypoxia-activated cancer therapy. Furthermore, we examined the efficacy of PTT/PDT therapy combined with hypoxia-activated chemotherapy. Firstly, the LC and LT IC50 (91.53 and 73.62 μg/ml, respectively) were obtained according to Figures S4, S5, and the ratio of LC and LT IC50 (5:4) was selected as the ratio of CyI and TPZ for the combination of LCT, then the cell survival rate of LCT was evaluated at different concentrations as shown in Figure 5(A), and the IC50 of CyI and TPZ in LCT was used to evaluate the combined effect.

Next, to evaluate the synergism of phototherapy induced by CyI and hypoxia-activated chemotherapy-induced by TPZ, the combined index (CI) of CyI and TPZ was calculated following Equation (3):

$$\mathrm{CI}\;=\;\mathrm{DA}/{\mathrm{IC}}_{50},\text{ A }+\;\mathrm{DB}/{\mathrm{IC}}_{50},\;B$$

where IC_50_, A and IC_50_, B are the concentrations of CyI and TPZ when they are used alone to reach the growth inhibition rate to X, respectively; DA and DB are the concentrations of CyI and TPZ when the growth inhibition rate is up to X by the combination of two drugs. As shown in Figures S4, S5, the IC50 for the individual use of LT and LC was 73.62 and 91.53 μg/ml, respectively. When using LCT for combined treatment, the two different IC50 are presented for CyI and TPZ. As shown in Figure 5(A), the IC50 of CyI and TPZ has reduced to 30.69 and 38.3 μg/ml, respectively, and the CI value was calculated to be 0.8. It is known that when the value of CI is below 1, the combined effect of the two drugs indicates a synergistic effect, confirming that the combined phototherapy and the bioreductive chemotherapy had a synergistic effect, rather than simple superposition.

Then, *in vitro* antitumor efficacy of PBS, NIR light, TPZ, LT, LC, and LCT under different laser irradiations (dark, 0.3 or 0.96 W/cm^2^) was evaluated. As shown in Figure 5(B), the cells cultured in PBS or only with NIR light did not show significant cell death (12.74 ± 5.58% cell death for PBS, 15.34 ± 1.76% cell death for NIR irradiation only). In contrast, the survival rate of cells treated with LT (57.25 ± 2.54% cell death), and LCT without light irradiation (59.7 ± 3.27% cell death) was slightly lower than that of TPZ (53.4 ± 4.93% cell death), which may be because TPZ coated in the hydrophilic cavity of liposome could reduce the loss of drugs. Meanwhile, since CyI had no cytotoxicity in dark, the cell survival rate of LCT without light irradiation was similar to that of LT. Under NIR irradiation, the inhibition rate of LCT (78.1 ± 4.12% cell death) was much higher than that of LC (63.9 ± 5.84% cell death), even under lower laser power density (0.3 W/cm^2^), further demonstrating the synergistic effect of phototherapy and hypoxia-reduced chemotherapy. Consistent with our above results, LCT with higher laser power density (0.96 W/cm^2^) showed a higher inhibition rate (86.62 ± 0.96% cell death) that than with lower power density (0.3 W/cm^2^, 78.1 ± 4.12% cell death), which proved that PDT/PTT combined with hypoxia response chemotherapy showed the best therapeutic effect.

The synergistic effect was further proved via the fluorescence pictures of cells co-staining with AM/PI to quantitatively study the number of living and dead cells (Figure 5(C)). Similar results were obtained that after being treated with synergistic PTT/PDT/hypoxia therapy, cells were almost dead; while the control group showed less dead cells than phototherapy activated anoxic chemotherapy, which confirmed the significant phototoxicity of LCT under hypoxic environment. Cell viability was also accessed by flow cytometry analysis via annexin V-FITC/PI staining (Figure 5(D)). As shown, cells incubated with LCT showed considerably high cell apoptosis upon NIR light, with the cell death rate up to 94.10%, resulting from the therapeutic effect of PTT/PDT/hypoxia therapy.

### *In vivo* animal study

**Figure 6.**
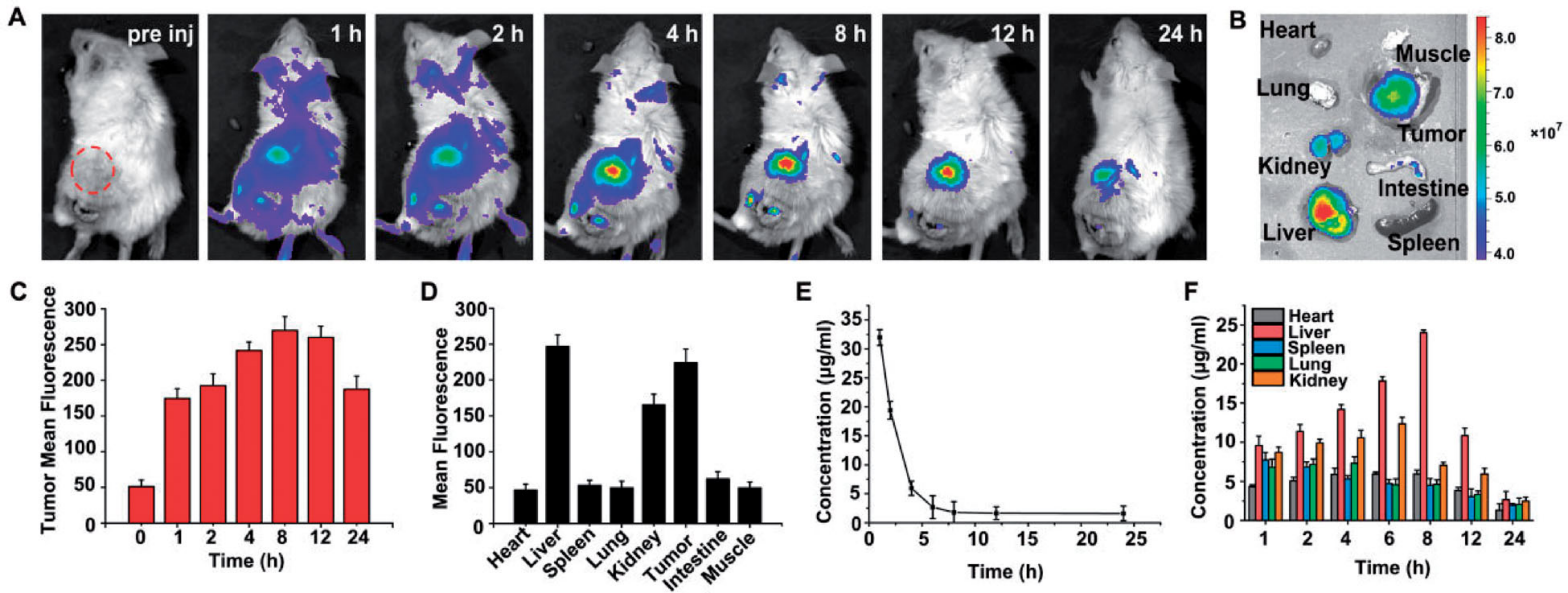
(A) *In vivo* dynamics of LCT in tumor-bearing mice; (B) NIR images of main organs collected from mice 24 h; (C) Quantitative fluorescence analysis of tumor at different time after injection via tail vein; (D) The fluorescence analysis of main organs at 24 h; (E) Plasma concentration curve of CyI after intravenous LCT (1.2 mg/kg equivalent to CyI) (*n* = 3); (F) *In vivo* tissue distribution of CyI (*n* = 3).

Before evaluating the *in vivo* efficacy of LCT, the distribution and pharmacokinetics of LCT in mice were firstly studied. As shown in Figure 6(A), the dynamic and tissue distribution of CyI in the LCT was monitored by the NIR imaging system. The fluorescence spread over the whole body after 1 h post-injection, and the tumors were identified after 4 h and maintained up to 24 h. This can be explained that in the solid tumor tissue, there are abundant blood vessels, a wide vascular wall gap, and a lack of lymphatic reflux, which result in the high permeability and retention of LCT, thus allowing more accumulation of LCT to the tumor tissues. The mice were sacrificed at 24 h post-injection and the main organs were isolated for NIR fluorescence imaging (Figure 6(B)). As shown, apart from the tumor tissues, the fluorescence was mainly concentrated in the liver and kidney, indicating that LCT was mainly eliminated through liver metabolism and kidney excretion (Monteiro et al., 2018). No significant fluorescence signals were found in the heart, lung, spleen, muscle, and small intestine, and the quantitative analysis of tumor and other organs distribution also confirmed these results (Figure 6(C,D)), suggesting that the LCT have a high level of tumor accumulation and good biological safety.

Next, the pharmacokinetics in plasma and tissues were further studied. The curve of average plasma concentration in Figure 6(E) and S11A showed that CyI and TPZ were not discovered in plasma after 6 h post-injection, exhibiting that LCT could be quickly removed from the plasma. The analysis of tissue distribution (Figure 6(F) and Figure S11(B)) indicated CyI and TPZ firstly gathered in the liver and kidney after intravenous injection, and reached a peak at 8 and 6 h, respectively; and then can be cleared from the mice after 24 h, confirmed that LCT was mainly metabolized by liver and kidney. It should be noted that the PK and biodistribution of TPZ were similar to that of CyI, further confirming that uploaded drugs (CyI and TPZ) were stable in LCT before light irradiation, in correspondence with the results of the release profile in Figure 1(G). Moreover, to further evaluate the safety of LCT, we conducted a hemolysis study. Triton-X is a kind of cell membrane breaker, which is used as the control of the hemolytic rate at 100%. Results in Figure S6 showed that even at the highest LCT concentration (500 μg/ml), the hemolysis rate is lower than 5%, which proved that LCT has good biocompatibility.

**Figure 7.**
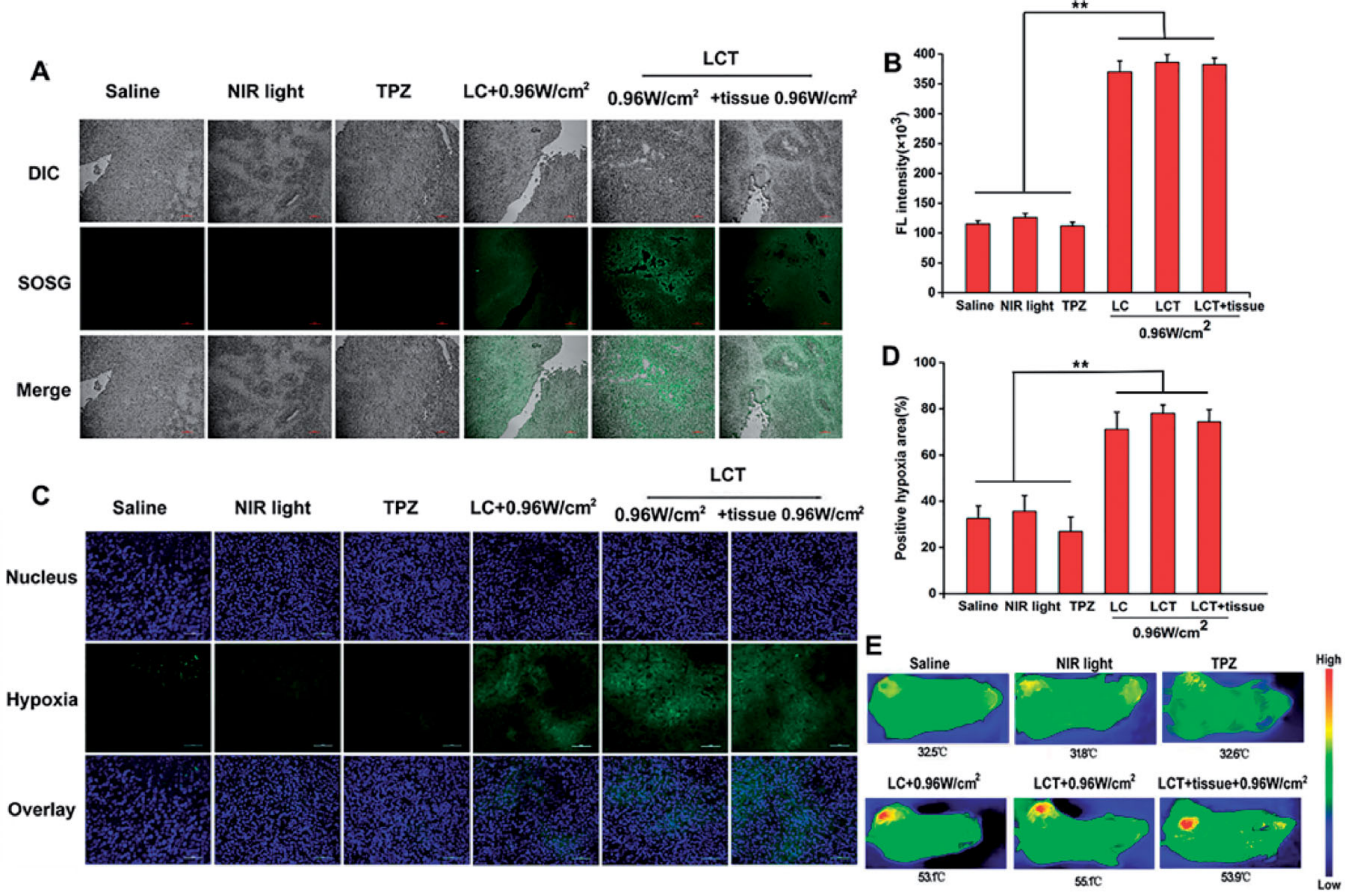
(A) The confocal fluorescence pictures of SOSG-stained slices at 6 h during various treatments (Scale bar = 100 µm); (B) Quantification fluorescence intensity of ^1^O_2_ production (*n* = 3). **p < .01; (C) Immunofluorescence pictures of tumor slices via anoxia staining (Scale bar = 100 µm); (D) Quantification fluorescence intensity of hypoxia area (*n* = 3). **p < .01; (E) Thermal images of 4T1 tumor-bearing mice at NIR light with various treatments.

The result of animal experimental treatment was shown in Figure 7. Firstly, to verify that PDT effect can be activated by NIR and produce ^1^O_2_ in tumor tissue, SOSG fluorescence probe was used. As shown in Figure 7(A), compared with saline, NIR light only and TPZ groups, LC and LCT group under NIR irradiation of 0.96 W/cm^2^showed obvious green fluorescence, demonstrating that a large amount of ^1^O_2_ was produced from CyI in liposomes. At the same time, we mimic the deep tissue group by adding a 1 cm pork tissue on tumors. As expected, the fluorescence intensity showed no significant difference from that of the LC and LCT, proving the strong penetration ability of near-infrared light. Semi-quantitative fluorescence analysis shown in Figure 7(B) confirmed that LCT could play an excellent PDT effect even in deep tissue under NIR light.

To verify that PDT consumes oxygen in the process of ^1^O_2_ production and provides a hypoxic microenvironment, the 4T1 tumor-bearing mice were subjected to immunofluorescent staining by a Hypoxyprobe-1 (Pimonidazole), which uses the activity of nitroreductase in anoxic tissue to convert the nitro group into amino group and hydroxylamine to detect hypoxia conditions inside the tumor. After the injection for 12 h, the tumor site was irradiated with a laser (0.96 W/cm^2^) for 5 min and then carried out for immunofluorescence detection. The hypoxia zone and the nucleus were stained with antipimonidazole antibody (green) and DAPI (blue), respectively; and the anoxic region of the tumor was quantified by semi-quantitative analysis. As shown in Figure 7(C,D), compared with the untreated only NIR light and TPZ group, the LC and LCT group under 0.96 W/cm^2^laser irradiation showed significant green fluorescence in the tumor region, indicating that the hypoxia was aggravated. Photothermal effects of LCT inside the tumor were also explored. The temperature change curves in tumors were shown in Figure S7. The temperature of tumor sites in LC, LCT, and LCT + tissue-treated groups increased to more than 50 °C under 0.96 W/cm^2^laser irradiation, which was much higher than that of photothermal treatment temperature (43 °C). Meanwhile, the maximum temperature of the tumor site was recorded by a thermal imaging camera (Figure 7(E)), and the results were consistent with that in Figure S7. These results proved that the CyI-mediated PDT/PTT could promote the production of ^1^O_2_ and heat generation, leading to aggravated hypoxia of tumor sites to stimulate biological reducing drug TPZ to play an anti-tumor effect.

**Figure 8.**
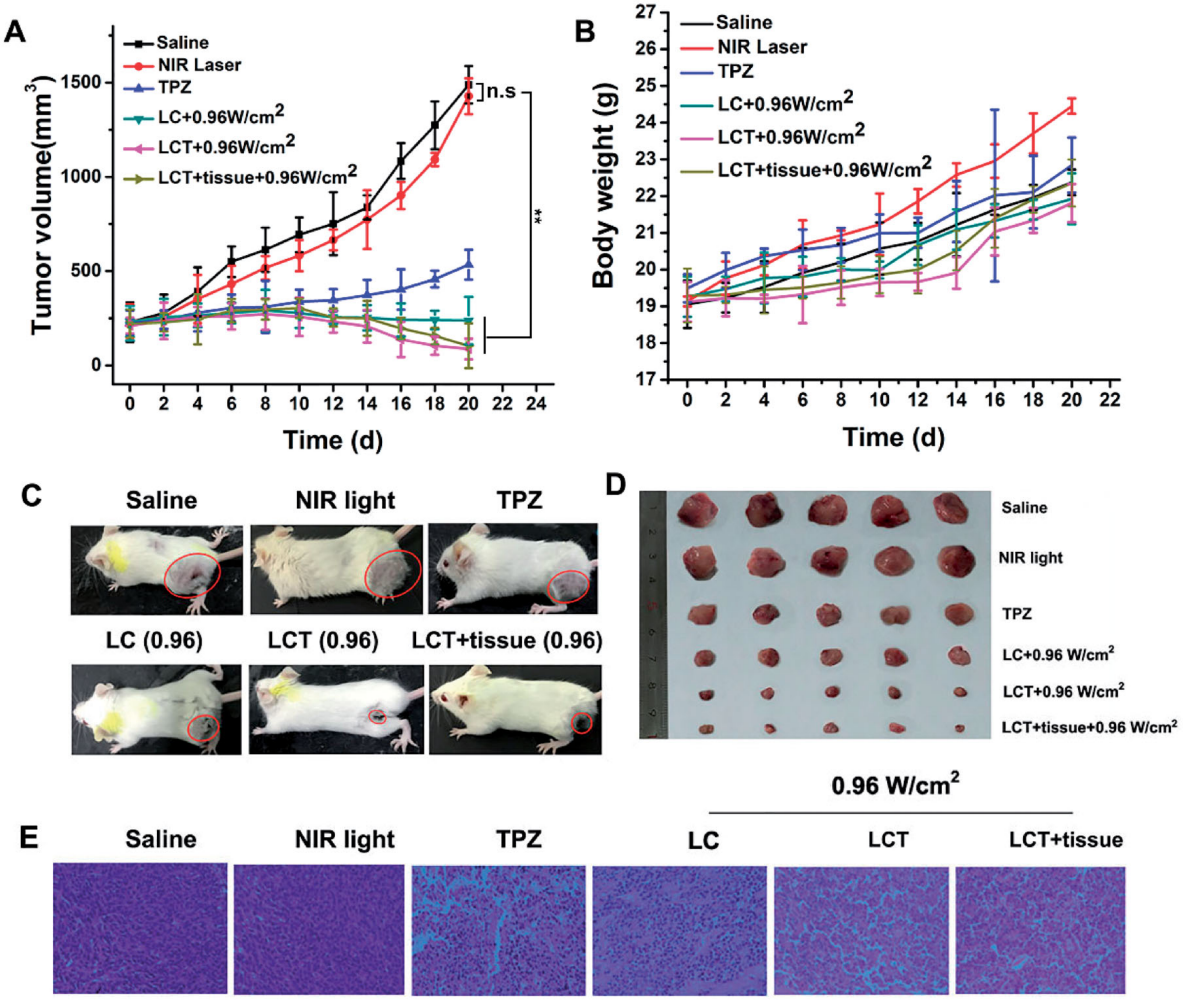
(A) Changes of tumor volume in mice during different treatments (*n* = 3). **p < .01, n.s: not significant; (B) The weight curves of mice after various treatments; (C) The photos of mice and excised tumors; (D) after various treatments. The red circles exhibit the tumor size; (E) The pictures of H&E stained tumor sections during different treatments (image magnification is 200×). The doses of CyI and TPZ in all the above experiments were 1.2 mg/kg CyI and 0.96 mg/kg TPZ.

Motivated by the effectiveness of PDT/PTT and PDT-induced hypoxia by LCT, we next investigate the synergistic therapeutic efficacy using the 4T1 subcutaneous tumor model. When the volume of the tumor increased to 250–300 mm^3^, all the mice were randomly separated into six groups and injected (i) saline; (ii) NIR light; (iii) free TPZ; (iv) LC + 0.96 W/cm^2^ irradiation; (v) LCT + 0.96 W/cm^2^ irradiation and (vi) LCT + tissue + 0.96 W/cm^2^ irradiation, respectively. The relative tumor volume of mice was monitored by vernier caliper every other day, and plotted in Figure 8(A). For the saline and NIR light groups, the tumor volume was rapidly increased, and no significant difference was found in the two groups, indicating that only application of light is harmless. The tumor growth in the TPZ group was slightly increased, indicative that when used alone, the anti-tumor effect of TPZ is limited. In contrast, LC, LCT, and the LCT + tissue group exhibited significant tumor inhibition under the NIR irradiation, among which the LCT group showed the highest inhibition rate, proving the significant advantages of the CyI-mediated PDT/PTT combined with the hypoxia-activated TPZ chemotherapy, even in deep tissues. In addition, there was a slight increase in the weight of each group of mice (Figure 8(B)), which means the low toxicity of the material we selected. After 21 days of treatment, a representative mouse and the tumors isolated from mice were photographed, as shown in Figure 8(C,D). The results showed no difference with the results of relative tumor volume, which further proved the high efficiency of synergistic PDT/PTT combined with hypoxic chemotherapy. H&E staining of tumor sections collected from 21 days after treatment were also evaluated (Figure 8(E)). As shown, the nuclear lysis and tumor necrosis were visible in tumors with treatments of LCT plus NIR light, while less nuclear pyknosis and nuclear fragmentation were observed after a single treatment (TPZ, LC + NIR light). This further verified the effective tumor-killing ability of LCT from the perspective of histology.

**Figure 9.**
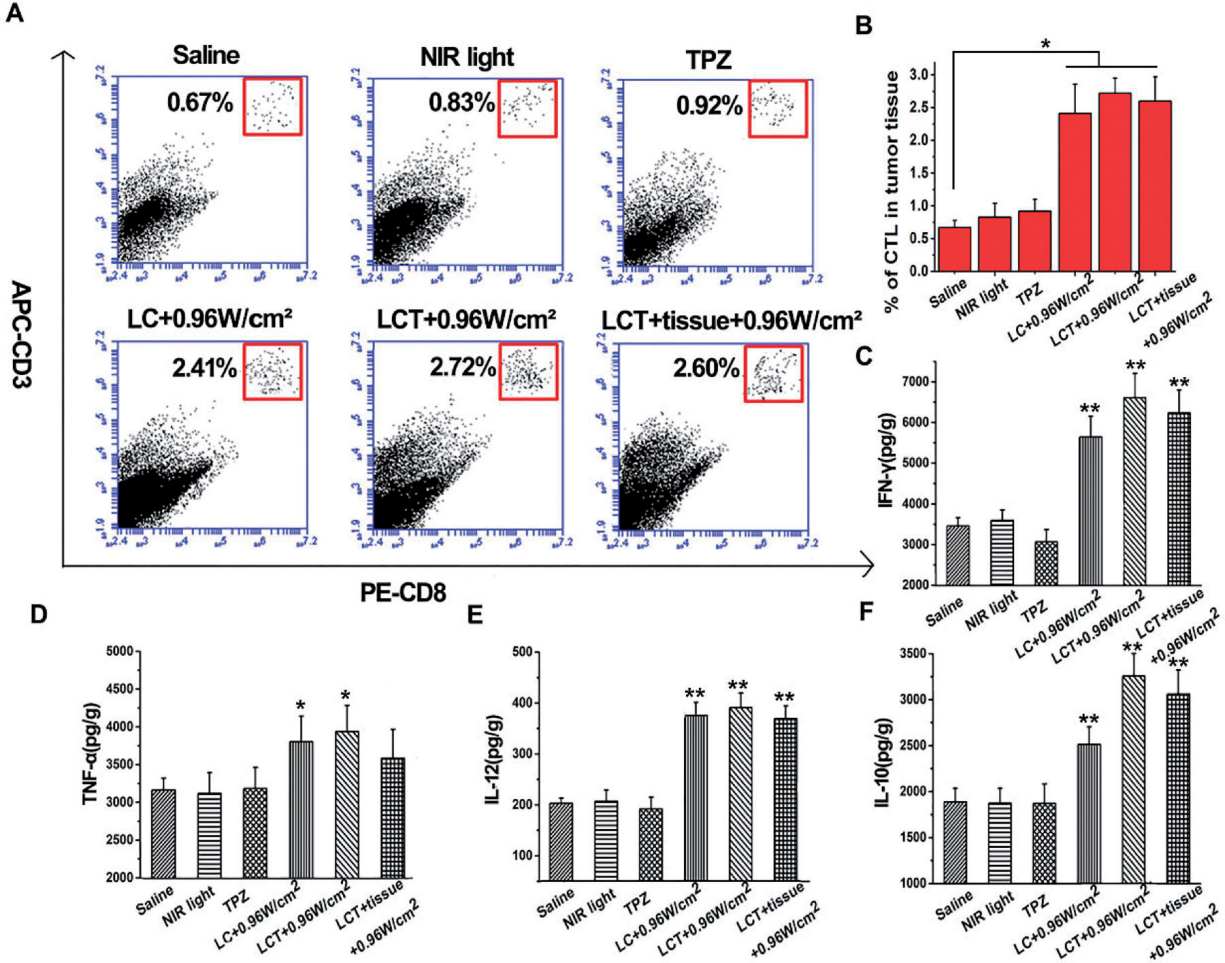
(A) The flow cytometry results of CTL infiltration in tumors; (B) Semiquantitative analysis of CTL (*n* = 3). *p < .05, **p < .01; (C–F) The immune factors concentration of IFN-γ (C), TNF-α (D), IL-12 (E), and IL-10 (F) in tumors (*n* = 3). *p < .05, **p < .01 *vs.* saline group.

It is reported that the ideal cancer treatment mode is to induce local tumor regression and eradication, as well as systemic anti-tumor immunity, which can effectively eradicate distant metastasis of cancer cells. To investigate whether LCT-mediated PDT could induce an immune response, the levels of immune factors IL-10, IL-12, TNF-α, IFN-γ in tumor tissues were detected by ELISA kit, and the content of Cytotoxic T lymphocyte (CTL) was quantified by flow cytometry. CTL is a kind of special T cell, which secretes various cytokines to participate in immune function. It can be seen from Figure 9(A,B) that the CTL expression of the CyI-mediated LC, LCT, and LCT + tissue was also significantly up-regulated after 0.96 W/cm^2^ laser irradiation, indicating that CD4^+^ helper T cells and CD8^+^ cytotoxic T cells activated to produce CTL, thus generating an immune response. IL-12 can induce the cytotoxic activity of CTL and NK cells and promote their secretion of IFN-γ, TNF-α, GM-CSF, and other cytokines, which play an important role in anti-tumor immunity. As shown in Figure 9(C–E), compared with the control group, the expression of IL-12, IFN-γ, and TNF-α in LC, LCT, and LCT + tissue groups were significantly up-regulated after 0.96 W/cm^2^ laser irradiation, indicating that PDT could induce the immune response of the body and play an anti-tumor effect. Interestingly, the expression of IL-10 was also up-regulated in the LC, LCT, and LCT + tissue groups (Figure 9(F)). As a cytokine leading to inflammation inhibition and immunosuppression, IL-10 can inhibit the synthesis of IL-12, reduce the amount of TNF-α and IFN -γ, and hinder the occurrence of the immune response. The up-regulation of IL-10 can be explained as its special physiological significance in limiting and preventing the body's excessive immune response to limit collateral damage. Meanwhile, this spontaneous immune response cannot prevent its anti-tumor immunity. These results suggest that LCT can be used as an effective immunomodulator for cancer treatment.

**Figure 10.**
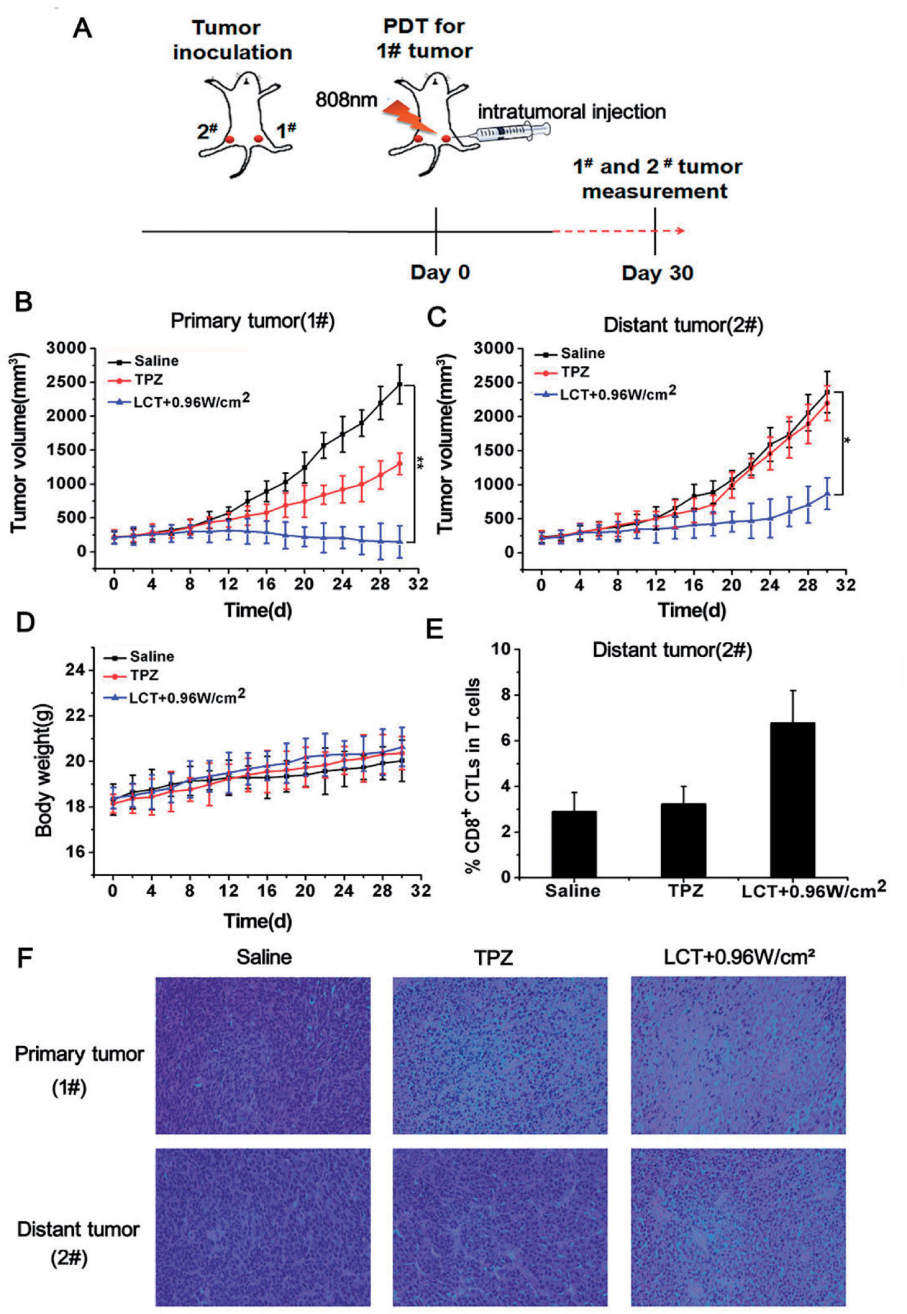
(A) Schematic illustration of our experiment design; Growth curves for primary tumors (B) and distant tumors (C) on mice after various treatments. (D) The weight curves of mice after various treatments (*n* = 3). *p < .05, **p < .01; (E) Proportions of tumor-infiltrating CD8^+^ T cells in the distant tumor collected on day 7; (F) The images of H&E for dual-tumor tissue slices of saline, TPZ and LCT groups (image magnification is 200×).

To further verify the systemic antitumor immune effect induced by PDT, we constructed a bilateral tumor model to examine the changes of the treated tumor and distant tumor. 10^6^ 4T1 tumor cells were subcutaneously inoculated into both the left and right flanks of each BABL/c mouse. The right tumor was designed as a primary tumor (1#) for basic PDT/PTT/chemotherapy, and the distant tumor in the left flanks (2#) was used as an untreated pseudo-tumor model for PDT mediated immune response (Figure 10(A)). When both tumors reached ∼100 mm^3^, we divided these mice into three groups: (1) Saline; (2) TPZ; (3) LCT + 0.96W/cm^2^, respectively. Fifty microliters of Saline, TPZ, and LCT (with CyI 1.2 mg/kg) were intratumorally injected into the right tumor tissue of each mouse, respectively, and the LCT group was irradiated with 808 nm near-infrared laser of 0.96 w/cm^2^ for 3 min. Subsequently, the dual-tumor volume and body weight were evaluated. As shown in Figure 10(B,C), for primary tumors, the LCT treatment group under 0.96 W/cm^2^ laser irradiation has the best tumor inhibition effect, while the use of chemotherapeutic drugs TPZ alone showed only moderate inhibition effect, which is consistent with our previous results. For distant tumors, LCT upon NIR irradiation showed a slight inhibition effect, partially delaying the growth of distant tumors, which was caused by PDT triggered systemic antitumor immune response, whereas groups treated with Saline and TPZ showed no significant immune response. These results suggest that after treatment with LCT, although the generated immune response cannot completely eliminate distant tumors, it can partially alleviate tumor growth. Besides, as shown in Figure 10(D), no significant changes in body weight were found, indicating that the construction of a dual-tumor model and the safety of the material was good. Next, we further investigated the mechanism of antitumor immunological responses post-PDT therapy. It is well-known that CD8^+^ T cells that bind to the antigen-MHC I complex could kill cancer cells by releasing the cytotoxins—IFN-γ, perforin, granzymes, and granulysin. Therefore, we collected the tumor on the left flank of the mouse on 7 days post-PDT to test the tumor-infiltrating CD8^+^ T cells by flow cytometry. The infiltration of CD8^+^ T cells in distant tumors increased after LCT treatment upon NIR irradiation (by ∼2-fold compared to the control). In contrast, no significant improvement of CD8^+^ T cell infiltration was observed in the Saline and TPZ groups (Figure 10(E)). Meanwhile, HE staining results also showed that for primary tumors, obvious nuclear cleavage and tumor necrosis could be found in LCT-treated tumors, and some degree of tissue damage and necrosis also appeared in distant tumors, which may be due to PDT triggered the systemic immune response. However, only primary tumors showed less tissue damage after TPZ treatment, and no tissue damage or obvious cell proliferation was found in distant tumors (Figure 10(F)). The above results show that the designed LCT liposomes can provide powerful systemic anti-tumor immunity to delay tumor growth after PDT treatment even without direct injection.

## Conclusion

A multifunctional liposome system co-encapsulating CyI as the photosensitizer and TPZ as the hypoxia-activated prodrug was designed for synergistic phototherapy and PDT-induced hypoxia-activated chemotherapy. This designed LCT could not only perform as a PTT/PDT/chemotherapy cascade-activated combination antitumor agent but also serve as an immunomodulator to induce secondary death of tumor cells. Because of tumor selectivity of TPZ and laser activated PDT, undesirable damage to normal cells could be effectively alleviated. Compared with conventional PDT or chemotherapy, LCT exhibited significantly improved anti-cancer efficacy. This liposome system overcame some of the key challenges in conventional tumor treatment and contributed great promise as a smart PDT/PTT/immune response and hypoxia-activated chemotherapy system for tumor diagnosis and therapy.

## Supplementary Material

Supplemental Material

## Data Availability

The data that support the findings of this study are available from the corresponding author upon reasonable request.
