# Supplementary material for: Iodinated cyanine dye-based nanosystem for synergistic phototherapy and hypoxia-activated bioreductive therapy
Source: Drug Deliv. 2022 Jan 8;29(1):238–53. doi: 10.1080/10717544.2021.2023701 (PMC8745379; doi:10.1080/10717544.2021.2023701)
Supplement: Supplemental Material [file IDRD_A_2023701_SM5391.docx]

**Iodinated Cyanine Dye-based Nanosystem for Synergistic Phototherapy and Hypoxia-activated Bioreductive Therapy**

Yunxia Dong *^a, #^*, Ling Zhou *^b, #^*, Zijun Shen *^a, #^*, Qingming Ma *^a^*, Yifan Zhao *^a^*, Yong Sun *^a^*^,^ *, Jie Cao *^a^*^,^ *

*^a^ Department of Pharmaceutics, School of Pharmacy, Qingdao University, Qingdao, China, 266021*

*^b^ The Key Laboratory of Traditional Chinese Medicine Prescription Effect and Clinical Evaluation of State Administration of Traditional Chinese Medicine, School of Pharmacy, Binzhou Medical University, Yantai, China, 264003*

*^#^ These authors contributed equally to this manuscript*

**Corresponding to* [*caojie0829@qdu.edu.cn*](mailto:caojie0829@qdu.edu.cn) *and sunyong@qdu.edu.cn*

**Supporting figures**


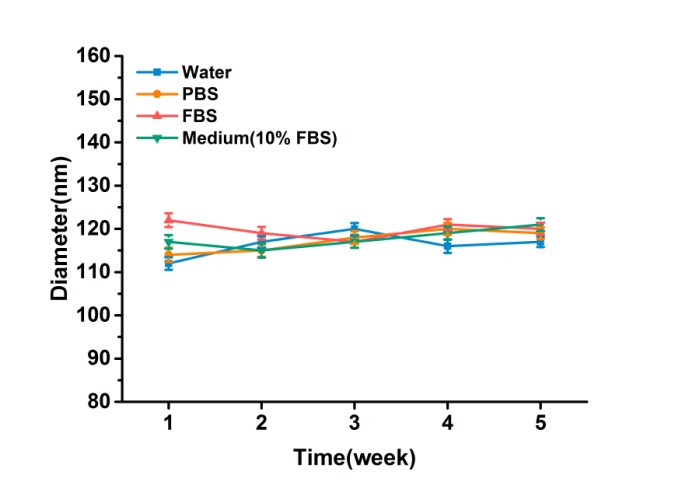


Figure S1 Size stability of LCT stored in water, PBS, FBS, and DMEM medium (10% FBS) in 5 weeks.


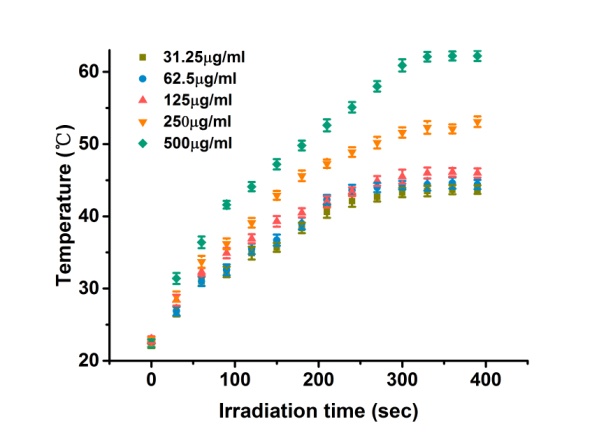


Figure S2 Temperature change curves of LCT aqueous solution with different concentration exposed to laser at a power density of 0.96 W/cm^2^.


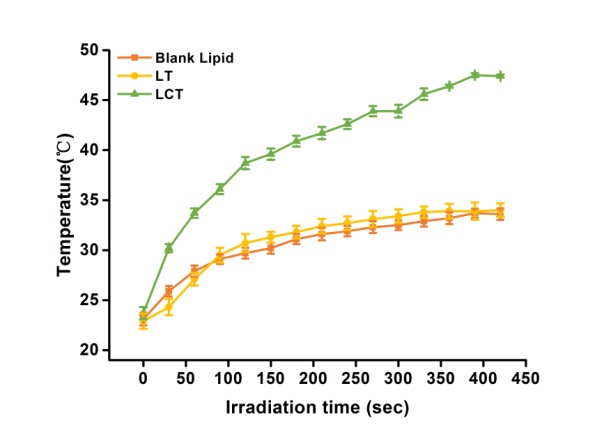


Figure S3 Temperature change curves of Blank Lipid、LT and LCT exposed to the 808 nm NIR laser at 0.96 W/cm^2^ power densities.


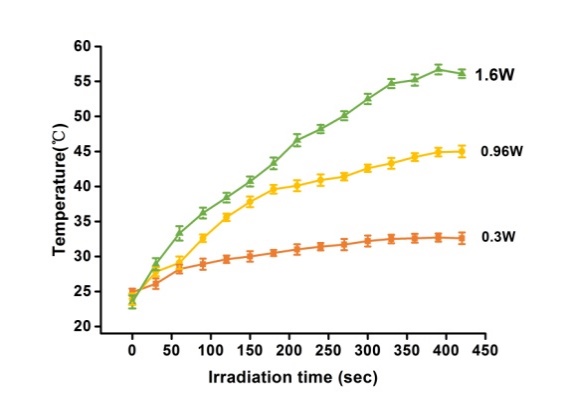


Figure S4 Temperature change curves of LCT exposed to the 808 nm NIR laser at different power densities (0.3, 0.96,1.6 W/cm^2^).


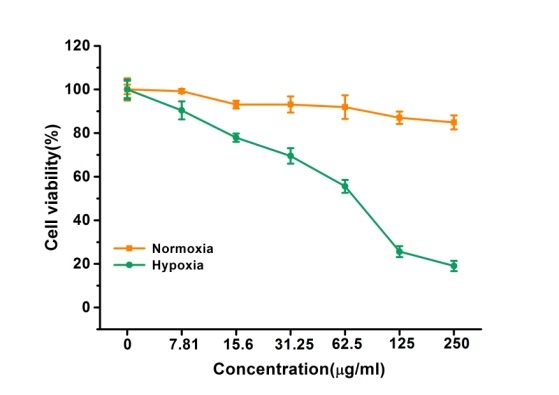


Figure S5 The cell viability after incubation with different concentration TPZ in hypoxic and normoxic atmosphere respectively.


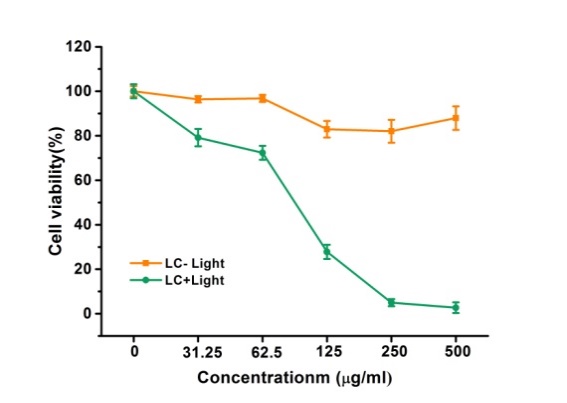


Figure S6 The cell viability of 4T1 cells after incubation with different concentration LC with or without 0.96W/cm^2^.


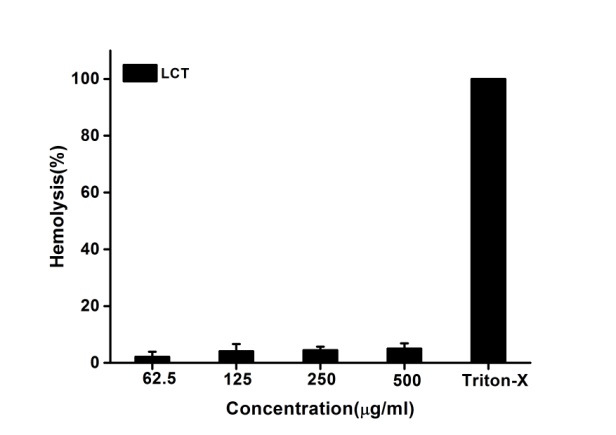


Figure S7 Hemolysis of LCT with different concentration (62.5,125, 250, 500μg/mL) in red blood corpuscle (RBC) suspension.


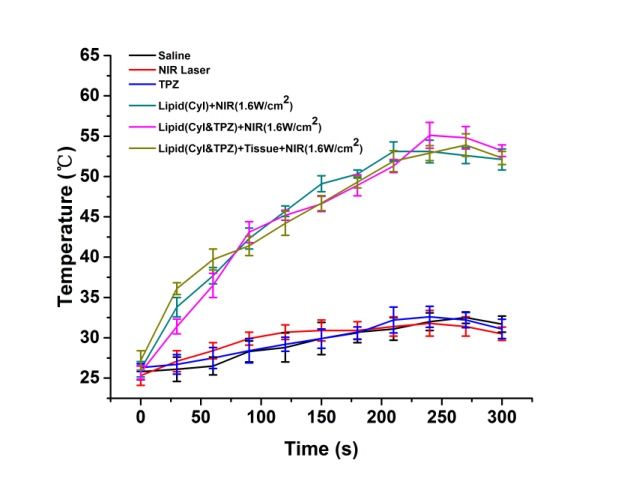


Figure S8 Temperature change curves in tumors exposed to the 808 nm laser at a power density of 0.96 W/cm^2^after intravenous injections of samples.

*
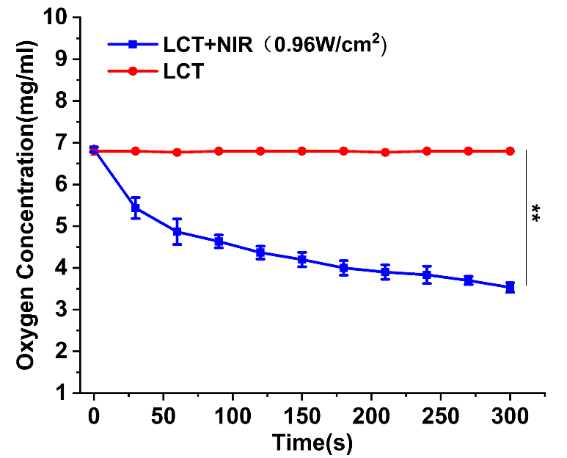
*

Figure S9. The oxygen concentration curve of LCT with or without laser irradiation (n=3). *p< 0.05, **p<0.01.


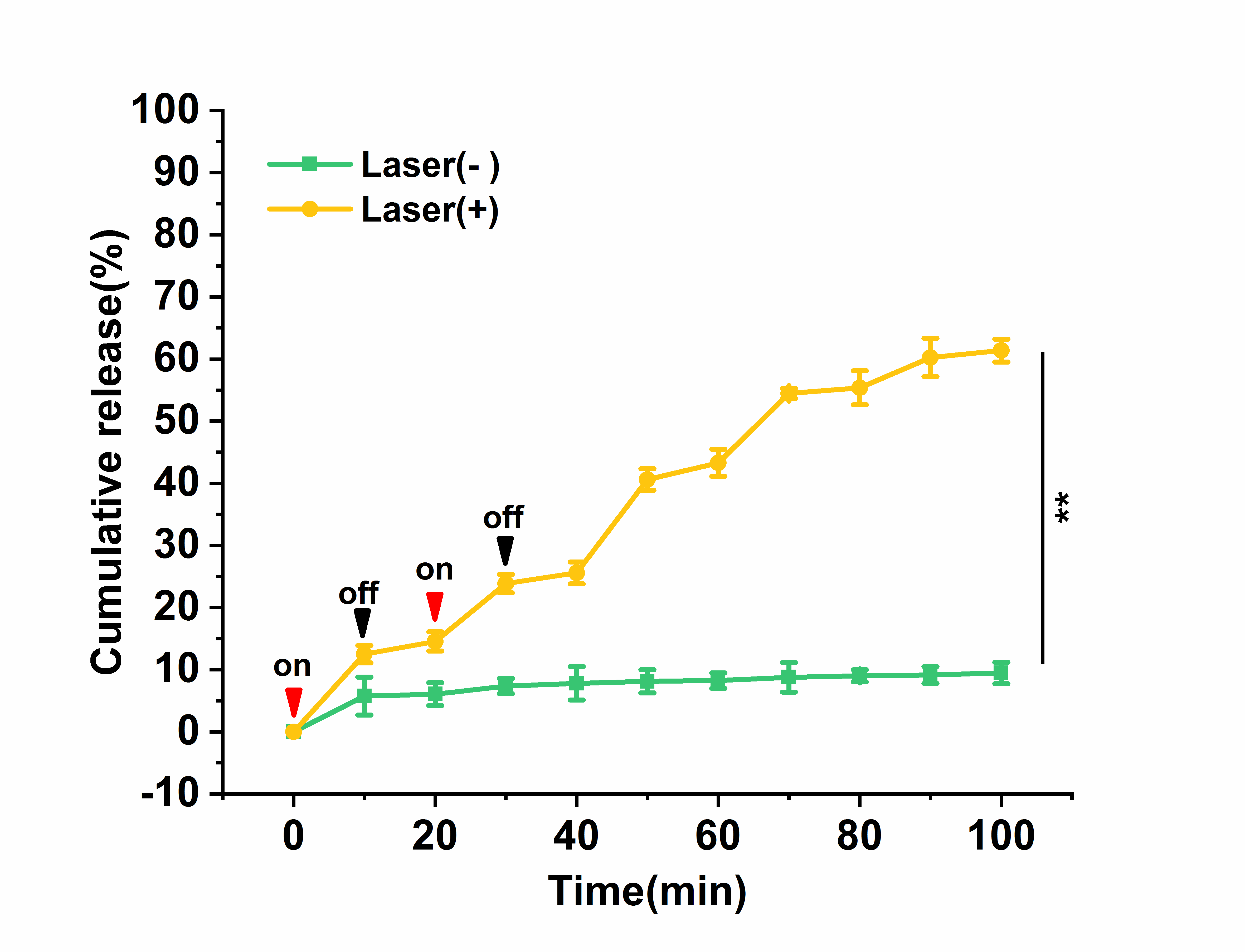


Figure S10. Cumulative CyI release curve from LCT with or without an “on-off” NIR light (0.96 W/cm^2^) for 100 min at 37 °C (n=3). *p< 0.05, **p<0.01.

*
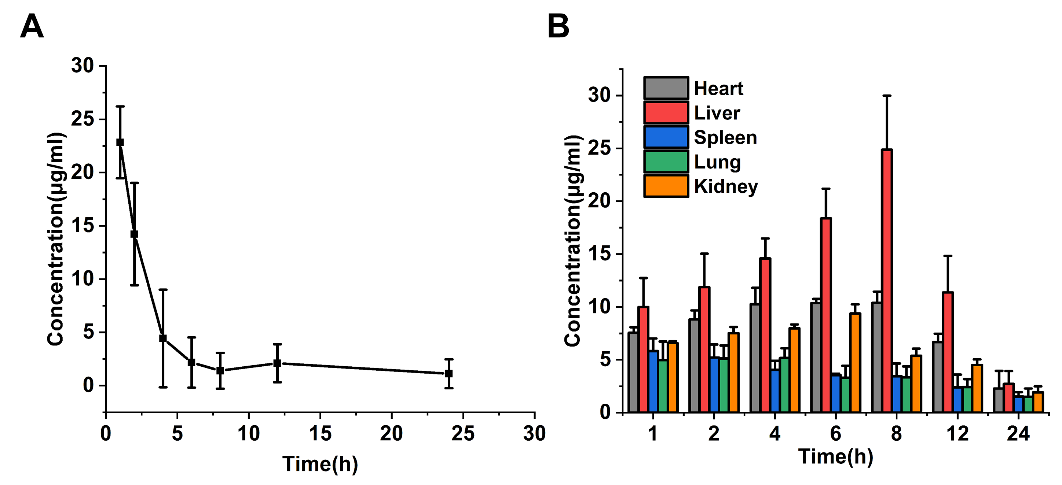
*

Figure S11. (A) Plasma concentration curve of TPZ after intravenous LCT (0.96 mg/kg equivalent to TPZ) (n=3); (B) *In vivo* tissue distribution of TPZ (n=3).
